# Anthrapyrazolone analogues intercept inflammatory JNK signals to moderate endotoxin induced septic shock

**DOI:** 10.1038/srep07214

**Published:** 2014-11-27

**Authors:** Karothu Durga Prasad, Jamma Trinath, Ansuman Biswas, Kanagaraj Sekar, Kithiganahalli N. Balaji, Tayur N. Guru Row

**Affiliations:** 1 Solid State and Structural Chemistry Unit, Indian Institute of Science, Bangalore, India; 2 Department of Microbiology and Cell Biology, Indian Institute of Science, Bangalore, India; 3 Department of Physics, Indian Institute of Science, Bangalore, India; 4 Supercomputer Education and Research Centre, Indian Institute of Science, Bangalore, India

## Abstract

Severe sepsis or septic shock is one of the rising causes for mortality worldwide representing nearly 10% of intensive care unit admissions. Susceptibility to sepsis is identified to be mediated by innate pattern recognition receptors and responsive signaling pathways of the host. The *c*-Jun N-terminal Kinase (JNK)-mediated signaling events play critical role in bacterial infection triggered multi-organ failure, cardiac dysfunction and mortality. In the context of kinase specificities, an extensive library of anthrapyrazolone analogues has been investigated for the selective inhibition of c-JNK and thereby to gain control over the inflammation associated risks. In our comprehensive biochemical characterization, it is observed that alkyl and halogen substitution on the periphery of anthrapyrazolone increases the binding potency of the inhibitors specifically towards JNK. Further, it is demonstrated that hydrophobic and hydrophilic interactions generated by these small molecules effectively block endotoxin-induced inflammatory genes expression in *in vitro* and septic shock *in vivo*, in a mouse model, with remarkable efficacies. Altogether, the obtained results rationalize the significance of the diversity oriented synthesis of small molecules for selective inhibition of JNK and their potential in the treatment of severe sepsis.

Sepsis, leading to organ dysfunction and hypotension unresponsive to fluid resuscitation, presents a significant challenge in preventive measures for mortality in hospital intensive care units (ICU). Sepsis causes millions of deaths every year worldwide^1^,^2^,^3^,^4^. Sepsis is often characterized by deregulated expression of wide-ranging host inflammatory genes leading to irreparable pathologies^5^. Integrated signaling pathways in host cells, which in turn tightly regulate transcriptional activation of inflammatory genes, frequently mediate these cellular events^6^. Owing to this sudden onset and excessive induction of inflammatory genes such as COX-2 and its catabolic end product PGE2 in addition to TNF-α, IL-12, IL-6, etc leads to multiple organ failure including vital organs like heart and central nervous system resulting in the death of individual^7^,^8^,^9^. It is observed that stress activated protein kinases (SAPKs) which comprise two main subsets, c-Jun N-terminal Kinase (JNK) and p38 MAPK (Mitogen Activated Protein Kinase), execute cell-fate decisions during sepsis^10^. SAPK/JNK family of kinases regulate downstream signaling events by controlled activation of c-Jun and c-Fos^11^,^12^. Phosphorylation of c-Jun by activated JNK at ser63 or ser73 promotes its interaction with c-Fos forming a functional transcription factor activator protein-1 (AP-1), a key regulator of pro-inflammatory genes expression (Fig. 1)^13^,^14^,^15^,^16^. AP-1 often acts as molecular switch in deciding cell-fate decisions across many cell types^17^. In this perspective, design and synthesis of highly potent target-specific inhibitors with limited or no off-target effects across cellular platforms assumes great importance to treat several disease-associated pathologies including sepsis^18^.

There are three isoforms of JNK: While JNK1 and JNK2 are found in all tissues, interestingly, JNK3 is localized to neuronal tissues, cardiac myocyte, and testes^19^,^20^. Thus, the distribution and expression patterns appear to contribute to the regulation of wide-ranging inflammatory immune responses which might also include sepsis. In this perspective, development of small molecule JNK inhibitors that are cell permeable, possessing rapid and reversible kinetics might provide powerful leads to develop therapeutics for the treatment of inflammatory disorders. Among the existing JNK selective inhibitors, SP600125 (anthra [1, 9] pyrazol-6 (2H)-one or 1,9-pyrazoloanthrone, Fig. 2), an anthrapyrazolone, is often utilized as a strong inhibitor of *c*-Jun N-terminal kinase catalytic activity. SP600125 inhibits JNK1, JNK2, and JNK3 with a high specificity and markedly inhibits the activation/phosphorylation of *c*-Jun as well as expression of inflammatory genes^21^. Despite its specificity, poor solubility in aqueous solutions and off-target effects on other kinases like monopolar spindle 1 (Mps1) has opened up new challenges in it's utility in treating severe sepsis^22^,^23^,^24^,^25^,^26^. The crystal structure of 1,9-pyrazoloanthrone (anthrapyrazolone) was found to display a positional disorder at the NH group of pyrazole ring and anthraquinone ring (Supplementary Fig. S1a) and its relevance in sensing specific anions has been traced to this disorder and more so to the acidity of the NH group^27^. Indeed the acidity of the NH group and pyrazole moiety of 1,9-pyrazoloanthrone is effective in forming specific hydrogen bonding at the ATP-biniding site of JNK. 1,9-pyrazoloanthrone forms hydrogen bonds with the carbonyl oxygen of Glu147 and the main chain nitrogen of Met149 in the enzyme-inhibitor complex (Protein Data Bank Code 1PMV) as shown in Fig. S1b-d, Supplementary Information^28^. As a consequence, several hydrophobic contacts with Ile70, Ala91, Met146, Leu148, Asp150, Asn152, Val196 and Leu206 develop at the active site of JNK.

Substitution of *N*-alkyl groups (methyl and ethyl) on 1,9-pyrazoloanthrone (anthrapyrazolone) resulted in the strengthening of hydrophobic interactions with the active site residues. However these experiments showed reduced inhibitory activity^21^. In a recent study, we have demonstrated the enhancement of hydrophobic interactions in two specific *N*-alkyl derivatives of 1,9-pyrazoloanthrone (propyl (SPP1) and butyl (SPB1)) with inhibition of *c*-JNK at lower concentration values <10 μM, considerably lesser than the concentrations required to inhibit *c*-JNK by 1,9-pyrazoloanthrone^29^. It was also shown by Brydon *et al.,* that substitution at the 7-position of 1,9-pyrazoloanthrone with a chlorine atom resulted in 2-fold improvement of inhibition^21^. These observations prompted us to explore an extensive library of 1,9-pyrazoloanthrone analogues (Fig. 2) with potent inhibitory capacities towards inhibition of JNK. In this study, rigorous evaluation of several anthrapyrazolone analogues has been carried out to assess the efficacy in terms of effects on expression profiles of inflammatory genes associated with septic shock both *in vitro* (in macrophages) and *in vivo* in murine model of sepsis. These analogues exhibited enhanced inhibitory activity over JNK (>10 fold than SP600125) with high selectivity and specificities. Altogether, we demonstrate the development and functional characterization of a series of specific compounds, which could act both as promising tools in elucidating the essential role of JNKs in cellular physiological processes as well as in the development of novel therapeutics to treat sepsis.

## Results

**Figure 2:**
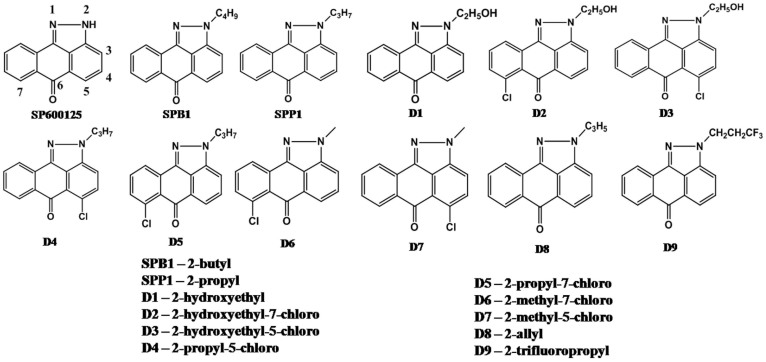
Molecular structures of the compounds and codes used. Numbering is given on SP600125 and is followed for analogues.

We have prepared a series of compounds based on anthrapyrazolone as a scaffold with different substitutions (Fig. 2) and the synthesis scheme is shown in Fig. S2 of Supplementary Information. The analogues were prepared as follows: (a) propyl (SPP1), butyl (SPB1) and allyl (D8) groups as N-alkyl substitution in anthrapyrazolone to activate hydrophobic contacts; (b) substitution of hydroxyethyl group (D1) supposed to activate hydrophilic contacts; (c) D4, D5, D6, D7 and D9 with alkyl groups and/or halogen atoms substituted on the periphery of anthrapyrazolone for enhanced hydrophilic contacts at the active site through halogen atoms and (d) D2 and D3 with both hydroxyethyl group and chlorine atoms which may provide additional hydrophilic interactions. These compounds were characterized by ^1^H and ^13^C NMR spectroscopy, ESI-MS and their purity is further confirmed by mass-directed preparative HPLC (analytical purity >99%). The analogues were crystallized from different solvents and their X-ray crystal structures were determined (Supplementary Fig. S3, Table S1). The selection of specific analogues of anthrapyrazolone for biochemical activity has been done as follows. The first step involves the analysis of docking features of each of the analogues followed by *in vitro* cell viability studies to benchmark the efficiency and specificity as well as cytotoxicity of each compound.

Docking simulations were performed with the coordinates taken from the PDB (Protein Data Bank) to evaluate the binding features. *In silico* studies were thus carried out using all three JNK structures viz. 1PMV^28^ (JNK3), 3E7O^30^ (JNK2) and 2NO3^31^ (JNK1). In addition, hydrophobic and hydrophilic interactions at the active site of JNK3 with SP600125 were also determined. The binding energy values obtained from docking studies which provide confirmatory evidence in terms of the best analogues of SP600125 (Table S2–S4, Supplementary Information). The binding energy of JNK3-SP600125 complex (PDB: 1PMV) is –8.05 Kcal mol^−1^. Molecules containing hydroxyethyl group with or without chloro group (D1, D2 and D3) and alkyl + chloro groups (D4, D5, D6 and D7) exhibited higher binding energies compared to the parent SP600125. This shows that substitution of hydroxyethyl/alkyl/chloro group on anthrapyrazolone should have improved the interactions leading to higher binding energies. On the other hand, allyl (D8) and trifluoro substitution in the alkyl chain (D9) did not show any improvement in the binding energy suggesting that such molecules are not suitable for stable productive interaction at the active site of JNK. The relative ranking of binding energies for the compounds is as follows: D1>D2>D3>D4>D5>D6>D7≈SP60015<D8<D9 (Table S2–S4, Supplementary Information).

Interestingly, *in silico* studies show that the analogues form both hydrogen and halogen bonds with active site residues in the binding site of 2NO3, 3E7O and 1PMV (Supplementary Fig. S4-S6). D1, D2 and D3 form hydrogen bonds with Met149 and Asn152 at the binding site of JNK3 and also D2 forms a halogen contact with Met149 in JNK3 active site (1PMV; Table S2, Supplementary Information).

### Anthrapyrazolone analogues inhibit JNK *in vitro*

**Figure 3:**
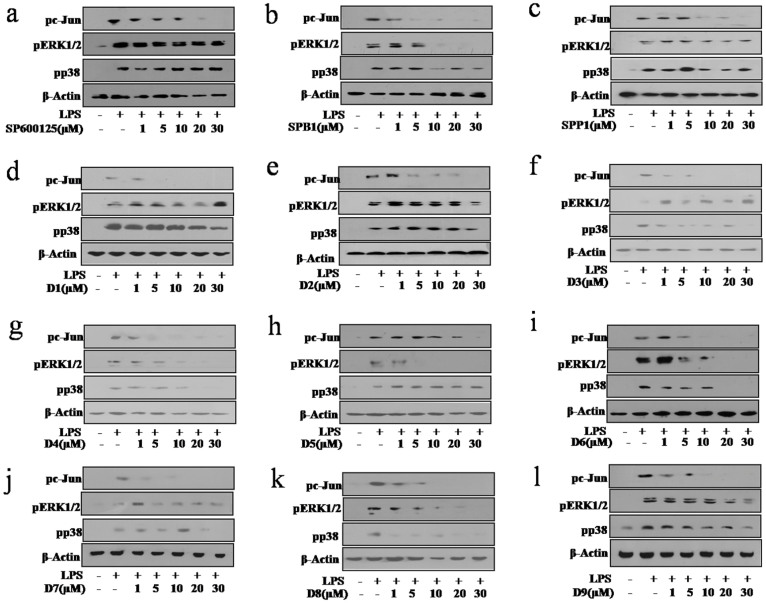
Treatment of mouse macrophages with anthrapyrazolone and its analogues reduces activation of MAPkinase along with JNK to a varied extent. Western blot analysis of pc-Jun in macrophages treated with respective inhibitors at higher concentrations as shown in (a) SPB1, (b) SPP1, (c) D9, (d) D1 (2-hydroxyethyl), (e) D2, (2-hydroxyethyl-7-chloro), (f) D3, (g) D4, (h) D5, (i) D6, (j) D7, (k) D8 and (l) SP600125 for 1 hour followed by LPS (100 ng/ml) treatment for additional 1 hour. Representative blots of three independent experiments are shown.

Cell viability assays coupled with western blot analysis of 1,9-pyrazoloanthrone (anthrapyrazolone) suggested that JNK activity is inhibited at concentration higher that 10 μM in LPS/endotoxin stimulated macrophages with minimal off-target effects on ERK1/2 and p38 MAPK (Supplementary Fig. S7a & S7b). Accordingly, minimal inhibitory concentration for 1,9-pyrazoloanthrone exceeded 10 μM in LPS triggered JNK mediated activation of AP1 promoter activity (Supplementary Fig. S7c). Prior to analysis of the inhibitory potentials of the analogues of 1,9-pyrazoloanthrone, a preliminary screen was carried out to examine the level of cytotoxicity on macrophages. In general, most of the tested analogues/molecules exhibited significant cytotoxicity (30%–40% decrease in cell viability) when used at concentrations >30 μM (Supplementary Fig. S8). This observation leads to the use of compounds at <30 μM concentration to assess their inhibitory efficacy on JNK in endotoxin treated macrophages. In addition to monitoring the specific inhibition of JNK, inhibitory properties of tested molecules were also evaluated on other MAPKs like ERK1/2 and p38 to assess their off-target effects (Fig. 3). Interestingly, D1 (2-hydroxyethyl) and D2 (2-hydroxyethyl-7-chloro) showed a significant selective inhibitory effect on JNK at concentrations ≤ 1 µM (Fig. 3d–e), which is 10 fold less as compared to SP600125 (Supplementary Fig. S7a & S7b) with minimal off-target effects on other MAPKs. Significantly, SPB1 (*N*-butyl derivative of 1,9-pyrazoloanthrone) exhibited potent inhibitory effects on JNK from 1 μM onwards. However, unlike D1 (2-hydroxyethyl) and D2 (2-hydroxyethyl-7-chloro), SPB1 demonstrated substantial off-targets effects on activation of ERK1/2 and p38 in macrophages (Fig. 3b). Similarly, SPP1 and D9 showed the inhibitory effect on JNK at concentrations as low as from 5 μM, but exhibited non-specific effects at higher concentrations (Fig. 3). This could be attributed to the varying binding energies exhibited by these compounds with that of JNK as well as for other MAPK's (Table S2–S4, Supplementary Information). It may be noted that compounds containing halogens (chlorine) may contribute to ligand interaction in forming steady halogen bonds with biological molecules. For example, D3, D4, D5, D6 and D7 exhibited potent inhibitory effect on JNK activity compared to SP600125 (Fig. 3f–k). However, despite their inhibitory effects at lower concentrations in comparison to SP600125, these inhibitors also blocked the activation of ERK1/2 and p38 MAPKs at higher concentrations, suggesting significant off-target effects (Fig. 3f–k).

**Figure 4:**
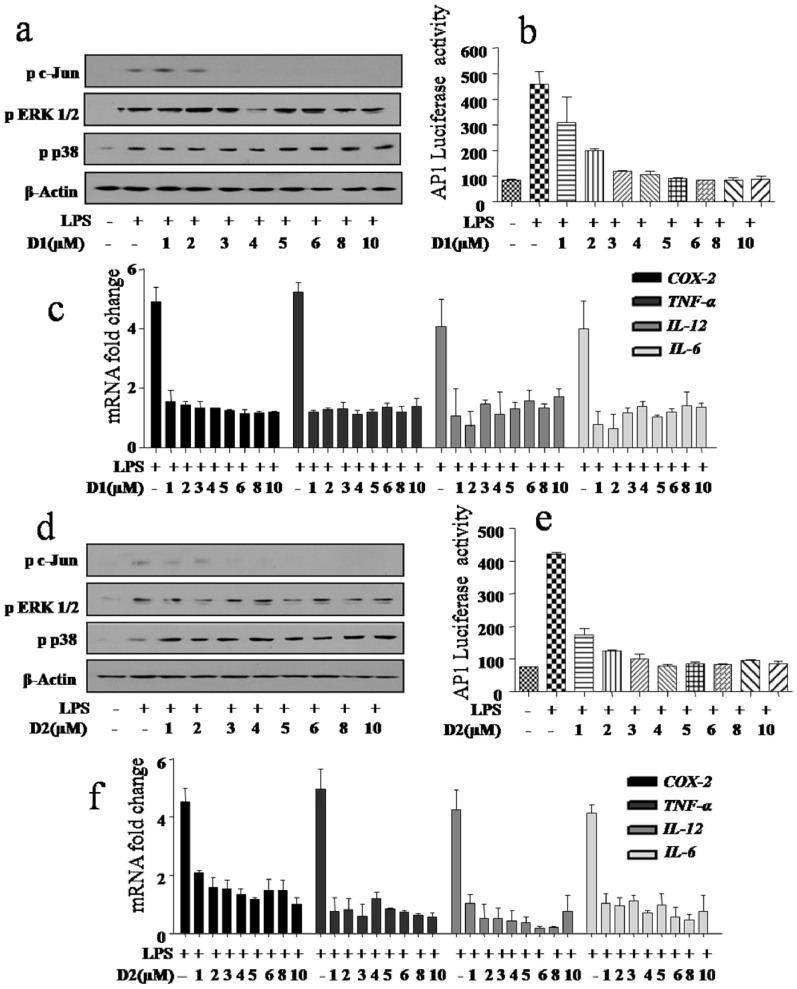
Anthrapyrazolone and its analogues inhibit JNK activity as well as other MAPkinases in concentration dependent manner in mouse macrophages. (a, b) Inhibitory effect of D1 (2-hydroxyethyl) over phosphorylation of c-Jun at lower concentrations analyzed by western blot in mouse macrophages and AP1 luciferase activity (RAW 264.7 macrophages) upon stimulation with LPS for 1 hour and 12 hour respectively. (d, e) Inhibitory effect of D2 (2-hydroxyethyl-7-chloro) over phosphorylation of c-Jun at lower concentrations analyzed by western blot and AP1 luciferase activity upon stimulation with LPS for 1 hour and 12 hour respectively. (c, f) Real Time PCR analysis of COX-2, TNF-α, IL-12 and IL-6 in macrophages upon treatment with D1 and D2 at lower concentrations in presence of LPS (100 ng/ml) respectively. Representative blots of three independent experiments were shown. n = 3, SE ± mean.

Of the molecules screened as summarized in Table 1, we attempted to elucidate the lowest possible concentration of D1, D2, D7 and D9 required to inhibit JNK activity *in vitro* as well as *in vivo*. Minimal inhibitory concentrations remain at 1 μM for D1 and D2 as analyzed by phosphorylation of c-Jun (Fig. 4) as well as JNK mediated activation of AP1 promoter activity (Fig. 4b & 4e) respectively. However, inhibition of JNK or JNK mediated activation of AP1 promoter activity required higher concentration of D7 and D9 (Supplementary Fig. S9). Further, at tested concentrations, D1 and D2 significantly inhibited the endotoxin-mediated expression of key immune genes including COX-2, TNF-α, IL-12 and IL-6 in macrophages (Fig. 4c & 4f). Interestingly, both D7 and D9 also inhibited endotoxin stimulated pro-inflammatory genes expressions (Supplementary Fig. S9). Taken together, these *in vitro* data clearly point out that D1 (2-hydroxyethyl) and D2 (2-hydroxyethyl-7-chloro) as potent inhibitors with greater selectivity in comparison to other inhibitors including commercially available JNK inhibitor SP600125 (anthrapyrazolone).

### Inhibitors of JNK block endotoxin triggered inflammation *in vivo*

**Figure 5:**
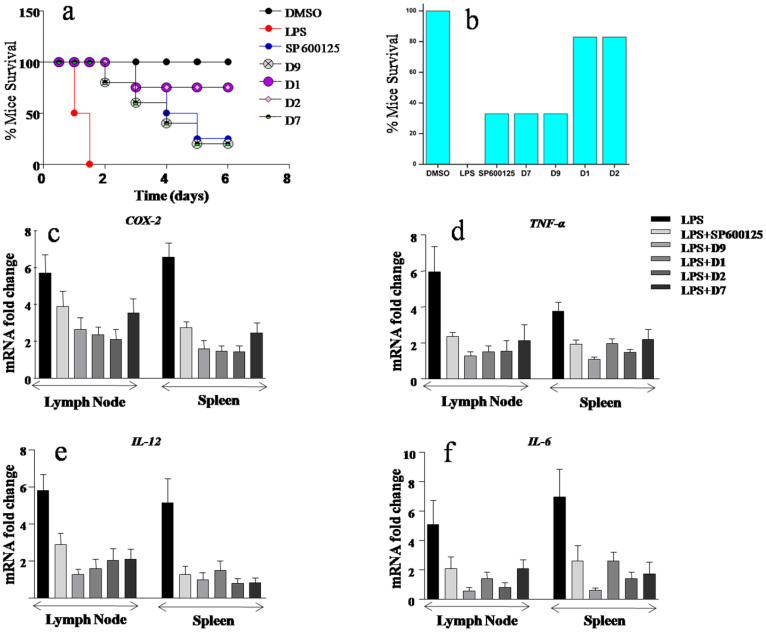
Anthrapyrazolone analogues block LPS induced inflammation *in vivo*. (a) LPS induced inflammation is inhibited by intravenous administration of the inhibitors as mentioned in materials and methods. Viability of mice is monitored at regular time intervals (no. of mice per each group = 6). (b) Bar graph representation of % mice survival data as given in (a). (c, d, e & f) Real Time PCR analysis of COX-2, TNF-α, IL-12 & IL-6 respectively in lymph nodes and spleen of mice injected with small molecule inhibitors. Representative blots of three independent experiments were shown. n = 3, SE ± mean.

Based on the inhibitory potential of a few selected molecules like D1, D2, D7 and D9, their efficacy in blocking as well as resolving endotoxin elicited inflammation in mouse model of septicemia was analyzed. Mice were intravenously administered with the selected molecules as described in materials and methods. Subsequently, mice were challenged intravenously with endotoxin and were monitored for their survival at regular intervals of 12 h. As shown, mice injected with endotoxin alone succumbed to death within 12 to 24 h. However, mice that were administered with D1 and D2 prior to endotoxin challenge exhibited significantly higher survival time. Interestingly, survival of mice that were administered with D7 and D9 prolonged compared to endotoxin challenged controls, matching the survival efficacy imparted by SP600125 (Fig. 5). DMSO, which serves as a vehicle control, did not show significant effects on the survival of mice throughout the chosen experimental time points (Fig. 5a–b). We do believe that considerable survival benefits imparted by D7 and D9 might be due to their off-targets effects on ERK1/2 and p38 MAPKs along with JNK. In all, it should be stressed that D1 and D2 mediated rescue of mice from endotoxin induced septic shock was significantly stronger compared to D7, D9 or SP600125.

Endotoxin mediated septicemia often involve excessive inflammation mediated by several inflammatory genes including COX-2, TNF-α, IL-12 and IL-6. As shown in Fig. 5c–f, D1 and D2 markedly reduced the endotoxin triggered COX-2, TNF-α, IL-12 and IL-6 expression levels in spleen and lymph nodes of endotoxin challenged mice which correlates with the survival of mice. Similar results were obtained with D7 and D9 (Fig. 5) but it should be remembered that the survivability of mice is shorter as compared to D1 and D2.

## Discussion

Endotoxin or LPS induced septic shock, is one of the serious concerns in ICU of hospitals where in microscopic level of contamination with hospital borne microbes culminates in massive induction of inflammation with augmented expression of several inflammatory molecules. We have screened a library of anthrapyrazolone analogues for their potential application in the treatment of Inflammation associated disorders such as sepsis. It is observed that hydroxyl and halogen substitutions on the periphery of 1,9-pyrazoloanthrone enhanced the binding potency towards JNK resulting in its inhibition. Based on the results from both *in vitro* with macrophages and *in vivo* with the mouse model of septicemia, the potential role of D1 and D2 in regulating endotoxin induced inflammation is firmly established. Altogether, the *in vitro* as well as the *in vivo* data clearly potentiates the selective inhibitory capacity of small molecule inhibitors like D1 and D2 which can facilitate the treatment of current inflammatory disorders when used in combination with the available drugs having varied efficacies. To our knowledge, this is the first description and rigorous biochemical characterization of selective JNK inhibitors. These inhibitors may facilitate the generation of novel therapeutics to treat sepsis and also pave the way to understand the essential biological functions of JNK.

## Methods

### Chemistry

Analogues of anthrapyrazolone were synthesized based on several modified procedures as described in the Supplementary Information details and provided synthetic schemes, procedures and additional characterization of these molecules.

### Isolation of peritoneal macrophages

The experiments with mouse macrophages were carried out after the approval from the Institutional Ethics Committee for animal experimentation as well as from Institutional Biosafety Committee. All the methods were approved by Indian Institute of Science and were carried out in accordance with the approved guidelines. Mouse peritoneal macrophages isolated from C57BL/6J were utilized for majority of the experiments in the current study. In brief, mice were intraperitoneally injected with thioglycollate (2 ml of 2X concentrate/mice). After 4 days of injection, mice were sacrificed and peritoneal cavity were flushed with ice cold PBS. Cells were pelleted down (1500 rpm/5 min at 4°C) and resuspended DMEM (Gibco, USA) supplemented with 10% fetal bovine serum for further experiments.

### Plasmids, transfections and inhibitor treatments

The AP1 luciferase construct used in the current study was assembled as described previously^32^. RAW264.7 macrophages were transfected with 7XAP1 luciferase construct with polyethylenimine (Sigma, USA) in serum free and antibiotic free medium. After 8 hours, medium was changed to complete DMEM and left for incubation for 48 hours. Later, macrophages were treated with LPS (100 ng/ml, Sigma, USA) in the presence or absence of respective inhibitors at various concentrations for 12 hours and promoter luciferase activity was analyzed. Small molecule inhibitors used in the study were added to the cells at respective concentrations 60 minutes prior to the treatment with LPS and then experiment was carried out for experimental time points. Inhibitors were retained till the end point of the experiment. All the inhibitors utilized in the study were dissolved in DMSO (Sigma, USA). 0.1% DMSO served as a vehicle control and had no effect on the LPS activated signaling intermediates in the current study.

### MTT assay

Mouse macrophages were seeded in 96 well plates (75,000cells/well) in 200 µl of DMEM complete medium and incubated overnight. Later cells were treated with various small molecule inhibitors reconstituted in DMSO at various concentrations as mentioned for 12 hours. Post 12 hours treatment, medium was removed carefully and fresh medium (100 µl/well) was added. 20 μl of MTT (5 mg/ml) reagent was added to each well and incubated for 4 hours at 37°C aseptically. Medium was removed and cells were added with DMSO. Absorbance of the solution was measured at 550 nm using an ELISA reader (Molecular Devices, USA).

### Luciferase assays

Luciferase activity was assayed using luciferase assay reagent (Promega). The results were normalized for transfection efficiencies by assay of β-galactosidase activity.

### Western blotting

Macrophages were treated with respective small molecule inhibitor as mentioned and then stimulated with LPS (Sigma-Aldrich, USA), 100 ng/ml, for additional 60 min. Cells were washed twice with PBS, scrapped off the culture dish and collected by centrifugation. Cell lysates were prepared in RIPA buffer constituting 50 mM Tris-HCl (pH 7.4), 1% NP-40, 0.25% Sodium deoxycholate, 150 mM NaCl, 1 mM EDTA, 1 mM PMSF, 1 μg/ml of each aprotinin, leupeptin, pepstatin, 1 mM Na_3_VO_4_, 1 mM NaF and incubated on ice for 30 min. Whole cell lysates were collected by centrifuging lysed cells at 13,000 g, 10 min at 4°C. Equal amount of protein from each cell lysate was subjected to SDS-PAGE and transferred onto PVDF membranes (Millipore, USA) by semidry western blotting (Bio-Rad, USA) method. Nonspecific binding was blocked with 5% nonfat dry milk powder in TBST (20 mM Tris-HCl (pH 7.4), 137 mM NaCl, and 0.1% Tween 20) for 60 min. The blots were probed with anti phospho Ser 63 c-Jun or anti-Thr180/Tyr182 phospho p38 MAPK, anti-Thr202/Tyr204 phospho ERK1/2, (Cell Signaling Technology, USA) for 12 hours at 4°C and then washed with TBST thrice followed by anti-rabbit IgG HRP conjugated secondary antibody (Jackson Immuno Research, USA) for 2 hours at 4°C. Blots were washed and developed using enhanced chemiluminescence detection system (Perkin Elmer, USA) as per manufacturer's instructions. Blots were probed with anti-β-actin HRP (Sigma-Aldrich, USA) to ensure equal loading of protein.

### Real Time PCR

Macrophages were treated with respective small molecule inhibitors followed by treatment with LPS (100 ng/ml) for 12 hours. Total cellular RNA from macrophages was isolated by TRI reagent (Sigma-Aldrich, USA). 1 μg of total RNA was converted into cDNA using First strand cDNA synthesis kit (Bioline, UK). Quantitative real time RT-PCR was performed using SYBR Green PCR mixture (KAPA Biosystems, USA) for quantification of the target gene expression. All the experiments were repeated at least three times independently to ensure the reproducibility of the results. In case of *in vivo* challenge studies, total RNA was isolated from the tissues and cDNA was synthesized as mentioned above and used for RT-PCR analysis of various target genes. The primers used for quantitative RT-PCR amplification are as follows: COX-2 FP: 5′- gtatcagaaccgcattgcctc-3′, COX-2 RP: 5′-cggcttccagtattgaggagaacagat-3′; TNF-α FP: 5′-agcccacgtcgtagcaaaccaccaa-3′, TNF-α RP: 5′-acacccattcccttcacagagcaat-3′′; Il-12 FP: 5′-gaagttcaacatcaagagcagtag-3′, IL-12 RP: 5′-agggagaagtaggaatgggg-3′; IL-6 FP: 5′- aaagagttgtgcaatggcaattct-3′, IL-6 RP: 5′-aagtgcatcatcgttgttcataca-3′. GAPDH FP: 5′-gagccaaacgggtcatcatct-3′, GAPDH RP: 5′-gaggggccatccacagtctt-3′.

### *In vivo* challenge of mice

In each group 6 mice (C57BL/6J) were used to assess the effect of small molecules in LPS induced inflammation. Mice were intravenously injected with 100 μg of respective small molecule inhibitors. After 6 hours of injection, mice were intravenously challenged with LPS (10 ug/mice) to induce inflammation and were monitored for survival. Spleen and lymph nodes were isolated and utilized for RT-PCR analysis of COX-2, TNF-α, IL-6 and IL-12. Equal volumes of DMSO, vehicle control showed no significant effect on the mice survival.

### Statistical analysis

Levels of significance for comparison between samples were determined by the Student t test distribution and one-way ANOVA. The data in the graphs are expressed as the mean ± SE and p values < 0.05 were defined as significant. Graphpad Prism 5.0 software (Graphpad software, USA) was used for all the statistical analysis.

## Author Contributions

The project was conceived by K.N.B. and T.N.G. Project executed by J.T. and K.D.P. Both K.S. and A.B. contributed to the insilico studies. K.D.P. carried out the synthesis and structural characterization of compound library. J.T. performed biological experiments. K.D.P., J.T., K.N.B. and T.N.G. wrote the manuscript, and all authors discussed the results and commented on the manuscript. J.T. and K.D.P. contributed equally as first authors (#).

## Supplementary Material

Supplementary Information Supplementary Information

## Figures and Tables

**Figure 1:**
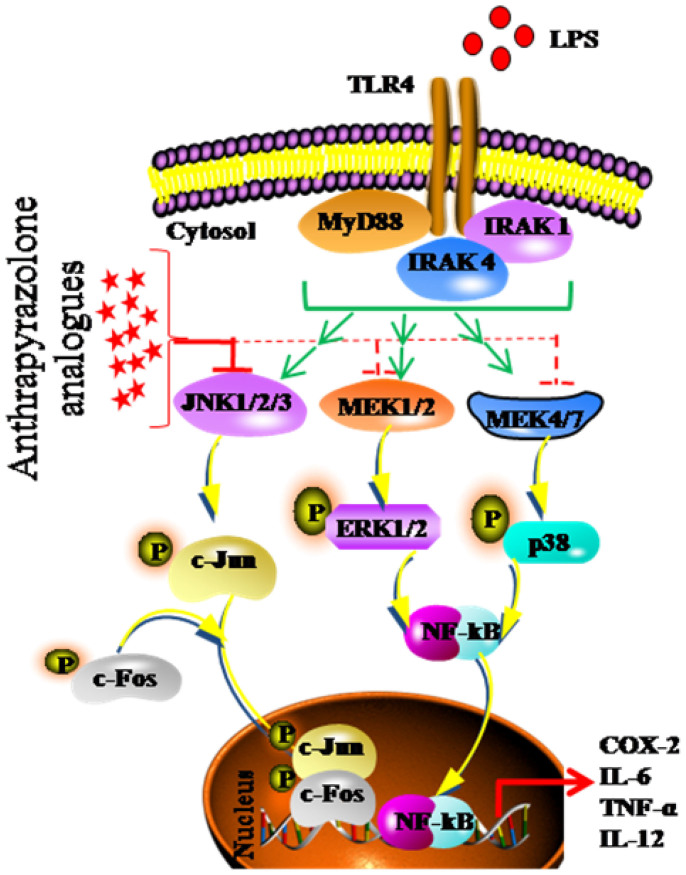
Endotoxin (LPS) mediated activation of JNK through Toll Like Receptor 4 (TLR4). Analogues of SP600125 or anthrapyrazolone inhibit JNK activity.

**Table 1:** List of inhibitors tested for *in vitro* analysis of JNK inhibition at different concentration

Inhibitors Tested	p c-Jun	p ERK 1/2	p P38
**SP600125**	>10 uM	No effect	No effect
**SPB1**	>5 uM	>10 uM	>10 uM
**SPP1**	>10 uM	No effect	No effect
**D1**	>1 uM	No effect	No effect
**D2**	>1 uM	No effect	No effect
**D3**	>5 uM	No effect	>5 uM
**D4**	>5 uM	>10 uM	>10 uM
**D5**	>20 uM	>5 uM	No effect
**D6**	>5 uM	>5 uM	>20 uM
**D7**	>1 uM	>5 uM	>20 uM
**D8**	>5 uM	>5 uM	>1 uM
**D9**	>1 uM	No Effect	>10 uM
