# Supplementary material for: Anthrapyrazolone analogues intercept inflammatory JNK signals to moderate endotoxin induced septic shock
Source: Sci Rep. 2014 Nov 27;4:7214. doi: 10.1038/srep07214 (PMC4245532; doi:10.1038/srep07214)
Supplement: Supplementary Information [file srep07214-s1.pdf]

Supplementary Information

Anthrapyrazolone analogues intercept inflammatory JNK signals to moderate endotoxin induced septic shock

Karothu Durga Prasad[1#], Jamma Trinath[2#], Ansuman Biswas[3], Kanagaraj Sekar[4], Kithiganahalli N. Balaji[2]* and Tayur N. Guru Row[1]*

[1]Solid State and Structural Chemistry Unit, Indian Institute of Science, Bangalore, India

[2]Department of Microbiology and Cell Biology, Indian Institute of Science, Bangalore, India

[3]Department of Physics, Indian Institute of Science, Bangalore, India

[4]Supercomputer Education and Research Centre, Indian Institute of Science, Bangalore, India

Corresponding Authors:

Tayur N. Guru Row, sscting@sscu.iisc.ernet.in, Fax: +91-80-23601310; Tel: +91-80-22932796

Kithiganahalli N. Balaji, balaji@mcbl.iisc.ernet.in, Fax: +91-80-23602697; Tel: +91-80-22933223

Table of Contents:

1. General Procedures: Page S2

2. Crystal structure of SP600125: Page S3

3. Synthetic Procedures: Pages S4 and S8

4. Crystallographic information of compounds: Pages S9-S10

5. Auto Docking studies (Binding energy tables for 3 JNK's): Pages S11-S12

6. Auto Docking studies (Lig plots for all molecules with 3 JNK's): Pages S13-S15

7. In vitro macrophage studies: Pages S16-S18

8. ^{1}H and ^{13}C NMR Spectra: Pages S19-S29

9. HPLC mass spectra: Pages S30-S33

10. References: Page S34

General Procedures: Column chromatography was performed on silica gel, Acme grade 100-200 mesh. TLC plates were visualized either with UV, in an iodine chamber, or with phosphomolybdic acid spray. All reagents were purchased from commercial sources and were used without additional purification. Unless stated otherwise, all reactions were performed under inert atmosphere. ^{1}H and ^{13}C NMR spectra were recorded on a 400 MHz machine in $CDCl_3$ as a solvent with TMS as reference. Chemical shifts are given in ppm with TMS as an internal reference. *J* values are given in hertz. HRMS was obtained using a micromass-QTOF spectrometer using electrospray ionization (ESI). Analytical HPLC analyses for all molecules was run on a Merck HPLC instrument (Auto sampler AS-2000, Interface Module D-6000, Pump L- 6200, Detector L-4250) equipped with a LiChrospher RP18 column (5µm) (Merck).

X-ray structure determination: Single crystal X-ray diffraction data were collected on an Oxford Xcalibur (Mova) diffractometer equipped with an EOS CCD detector using MoKα radiation (λ= 0.71073 A). The temperature on crystal was maintained using the Oxford Instruments Cryojet-HT controller during data collection. All structures were solved by direct methods using SHELXS-97 and refined against F2 using SHELXL-97.[1] H-atoms were located geometrically and refined isotropically. The WinGX[2] package and OLEX2 (version 1.2)[3] was used for refinement and production of data tables and ORTEP-3[4] for structure visualization and making the molecular representations. Analysis of the H-bonded and π•••π interactions was carried out using PLATON[5] for all the structures. Packing diagrams were analyzed by using MERCURY.[6]

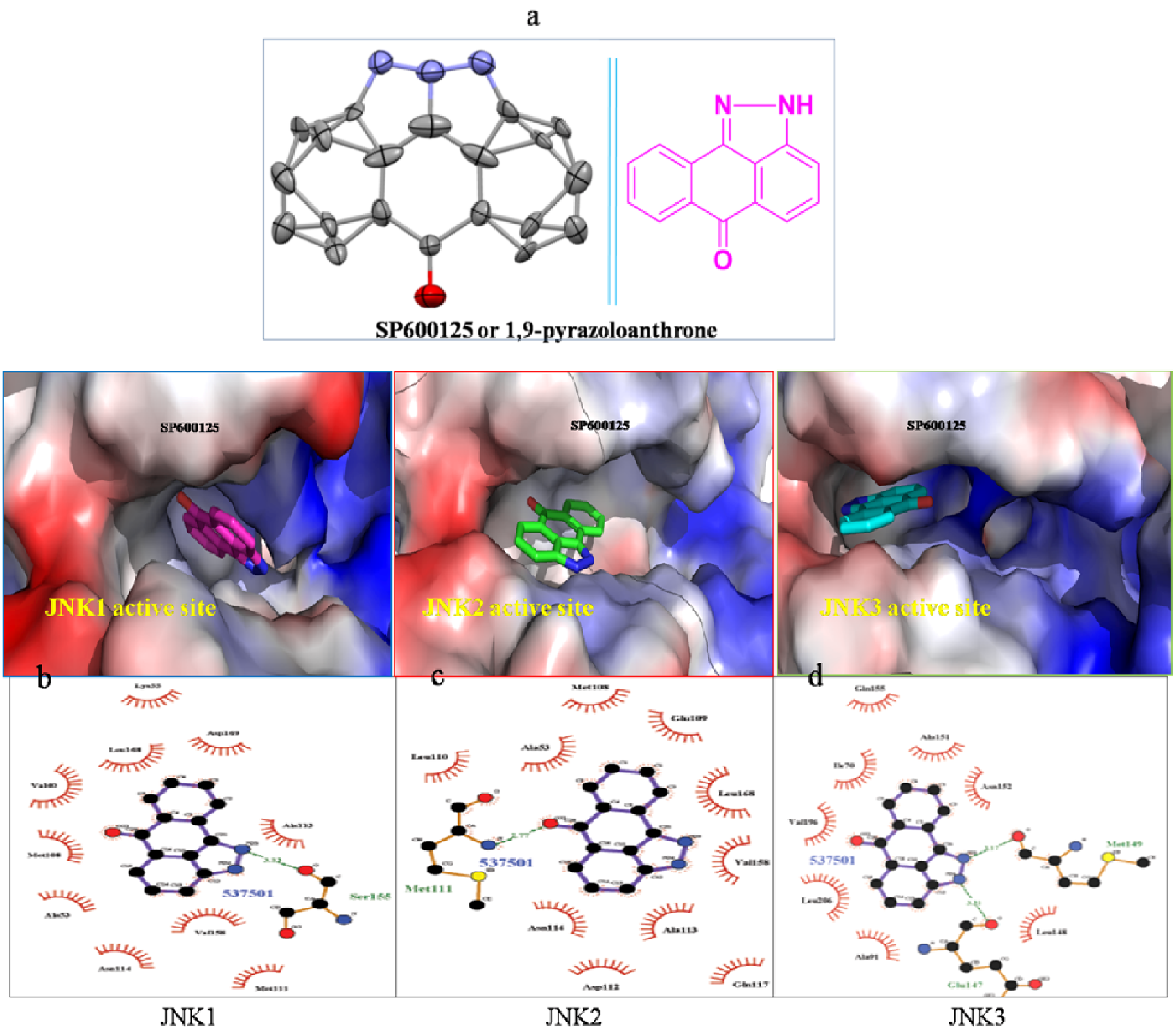

Figure S1 (a) Crystal structure of 1,9-pyrazoloanthrone (anthrapyrazolone). **(b, c, d)** 1,9-pyrazoloanthrone with the active sites of JNK 1, JNK 2 and JNK 3.

Synthesis of anthrapyrazolone analogues:

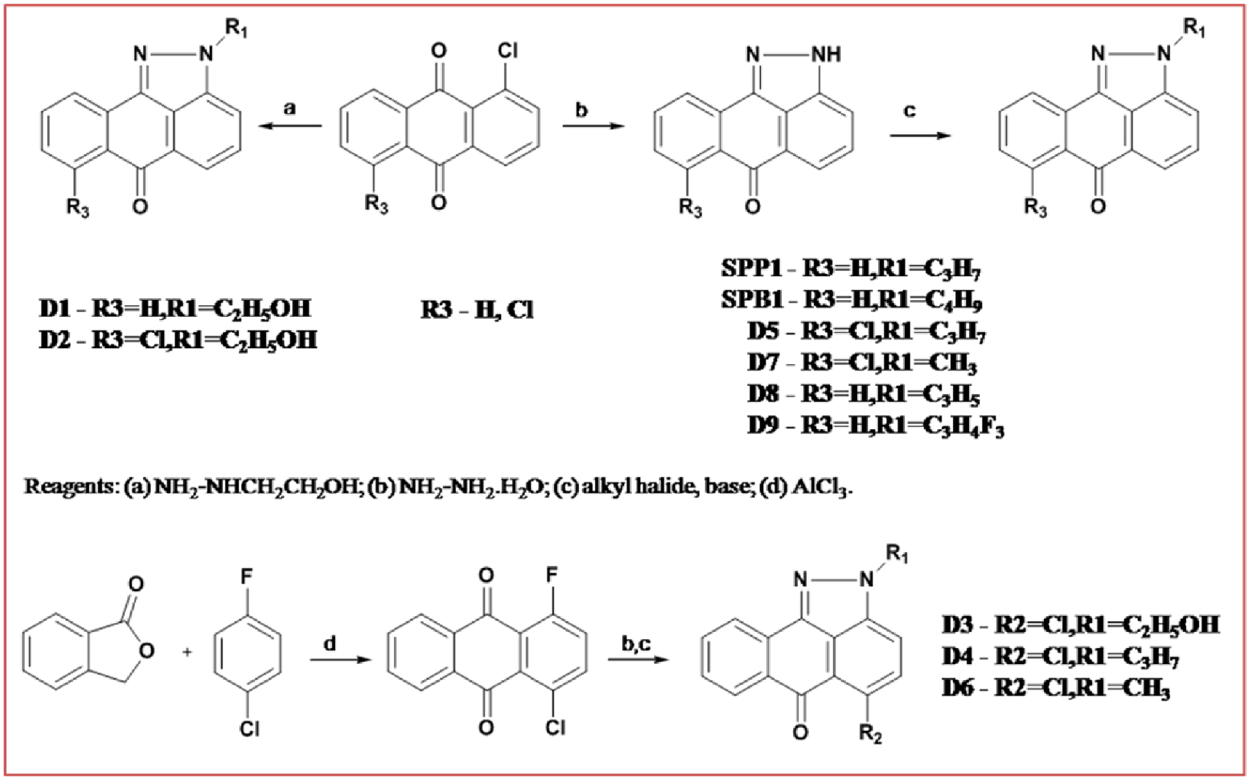

Figure S2 Represents scheme of synthesis of anthrapyrazolone analogues.

SP600125: HRMS for $C_{14}H_8N_2O$+Na calcd 243.0534; found 243.0538. HPLC: 99.20%, tR=4.97 min.

2-propyldibenzo[cd,g]indazol-6(2H)-one (SPP1): The compound was prepared according to modified procedure.[7,8] ^{1}H NMR (400 MHz, $CDCl_3$): δ 8.45 (d, J =7.52 Hz, 1H), 8.22 (d, J =7.64 Hz, 1H), 8.04 (d, J =6.84 Hz, 1H), 7.77-7.49 (m,3H), 7.51 (t, J =8.08 Hz, 1H), 4.49 (t, J =13.88 Hz, 2H), 2.15-1.99 (m,2H), 0.99 (t, J =14.72 Hz, 3H),; ^{13}C NMR (100 MHz, $CDCl_3$): δ 184.3, 139.6, 139.0, 133.7(2C), 132.4, 129.7, 128.6(2C), 126.9, 123.9, 123.0, 121.1, 115.4, 52.6, 24.3, 11.9.; HRMS for $C_{17}H_{14}N_2O$ +Na calcd 285.1004; found 285.1006.

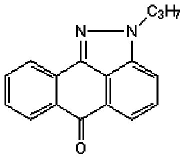

2-butyldibenzo[cd,g]indazol-6(2H)-one (SPB1): The compound was prepared similar to SPP1. ^{1}H NMR (400 MHz, $CDCl_3$): δ 8.44 (d, J =7.88 Hz, 1H), 8.21 (d, J =7.72 Hz, 1H), 8.02 (d, J =6.96 Hz, 1H), 7.75-7.57 (m, 3H), 7.52 (t, J =8.36 Hz, 1H), 4.51 (t, J =7.12 Hz, 2H), 2.00 (p, J =5.48 Hz, 2H), 1.76-1.28 (m,2H), 0.97 (t, J =7.4 Hz, 3H).; ^{13}C NMR (100 MHz, $CDCl_3$): δ 183.8, 139.1, 138.6, 133.2, 133.2, 132.0, 129.1(2C), 128.1, 126.4, 123.5, 122.6, 120.7, 114.9, 49.9, 32.4, 20.1, 13.7.; HRMS for $C_{18}H_{16}N_2O$ +Na calcd 299.1160; found 299.1158.

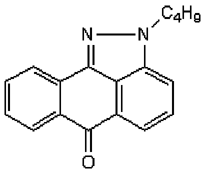

2-(2-hydroxyethyl)dibenzo[cd,g]indazol-6(2H)-one (D1): The compound was prepared according to the modified procedure.[9] Purification of this material is done by column chromatography (0.1% methanol in $CHCl_3$). Yellow color needle shaped crystals suitable for X-ray diffraction were obtained by slow evaporation of a mixture of $CHCl_3$ and DCM at ambient temperatures. Crystal structure was solved and refined in a triclinic space group P-1, Z=2. ^{1}H NMR (400 MHz, $CDCl_3$) δ 8.28 (d, J = 8.0 Hz, 1H), 8.02 (d, J = 7.6 Hz, 1H), 7.77 (d, J = 7.6 Hz, 1H), 7.66 (d, J = 8.0 Hz, 1H), 7.61 (t, J = 7.6 Hz, 1H), 7.55–7.41 (m, 1H), 4.60 (t, J = 4.8 Hz, 1H), 4.32–4.18 (m, 2H), 3.39 (t, J = 6.4 Hz, 1H).; ^{13}C NMR (100 MHz, $CDCl_3$): δ 183.8, 140.0, 139.3, 133.6, 133.2, 131.8, 129.3, 128.8, 128.7, 126.1, 123.3, 122.9, 121.2, 115.6, 62.6, 52.8; HRMS for $C_{16}H_{12}N_2O_2$ + Na calcd 287.0796; found 287.0793.

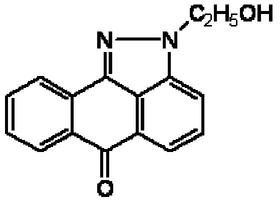

7-chloro-2-(2-hydroxyethyl)dibenzo[cd,g]indazol-6(2H)-one (D2): This compound was made using the same procedure as for D1 but 1,5-dichloroanthroquinone was used instead of 1-chloroanthroquinone. Purification of this material is done by column chromatography (1% methanol in $CHCl_3$). Brown yellow color needle shaped crystals suitable for X-ray diffraction were obtained by slow evaporation of $CHCl_3$/DCM mixture at ambient temperatures. D2 crystallized in a monoclinic space group $P2_1/c$, Z=4 with two molecules in the asymmetric unit; ^{1}H NMR (400, $CDCl_3$) δ 8.17 (d, J = 2.8 Hz, 1H), 7.98 (d, J = 7.2 Hz, 1H), 7.73 (d, J = 8.4 Hz, 1H), 7.65 (td, J = 7.6 , 1.2 Hz, 1H), 7.60-7.49 (m, 2H), 4.64 (t, J = 4.8 Hz, 2H), 4.30-4.16 (m, 2H), 2.74 (t, J = 6.0 Hz, 1H).; ^{13}C NMR (100 MHz, $CDCl_3$): δ 183.4, 139.6, 138.9, 133.2, 132.9, 129.0, 128.4, 128.2, 125.8, 123.0, 122.5, 120.8, 115.2, 62.1, 52.1.; HRMS for $C_{16}H_{11}ClN_2O_2$+ Na calcd 321.0407; found 321.0406 (M+Na).

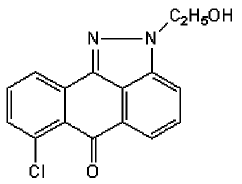

5-chloro-2-(2-hydroxyethyl)dibenzo[cd,g]indazol-6(2H)-one (D3): This compound was made by using the same procedure as for D2 but 1,4-dichloroanthroquinone was used. The compound was purified by column chromatography (0.1% methanol in $CHCl_3$). Brown yellow color needle shaped crystals suitable for X-ray diffraction were obtained from a mixture of solvents ($CHCl_3$/DCM/methanol) at ambient temperatures. Single crystal data were collected and refined in a Orthorhombic space group $Pca2_1$, Z=8 with two molecules in the asymmetric unit (Z'=2); ^{1}H NMR (400, $CDCl_3$) δ 8.34 (d, J = 8.0 Hz, 1H), 8.04 (d, J = 8.0 Hz, 1H), 7.70-7.57 (m, 2H), 7.52 (td, J = 8.0, 1.2 Hz, 1H), 7.46 (d, J = 8.0 Hz, 1H), 4.62 (t, J = 4.8 Hz, 2H), 4.33-4.20 (m, 2H), 3.19 (t, J = 6.4 Hz, 1H).; ^{13}C NMR (100 MHz, $CDCl_3$): δ 184.2, 133.8, 133.5 (2C), 131.9, 131.1 (2C), 129.6 (2C), 129.0, 122.8 (2C), 116.3, 62.5, 52.8.; HRMS for $C_{16}H_{11}ClN_2O_2$+Na calcd 321.0442; found 321.0450(M+Na).

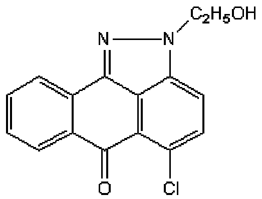

5-chloro-2-propyldibenzo[cd,g]indazol-6(2H)-one (D4): This compound was made by using the same procedure as for D3. The compound is purified by column chromatography (0.1% methanol in $CHCl_3$). Yellow color needle shaped crystals suitable for X-ray diffraction were obtained from a mixture of solvents ($CHCl_3$/DCM) at ambient temperatures. Single crystal data were solved and refined in a Orthorhombic noncentrosymmetric space group $P2_12_12_1$, Z=4, with one molecule in asymmetric unit (Z'=1); ^{1}H NMR (400, $CDCl_3$) δ 8.46 (d, J = 8.0 Hz, 1H), 8.20 (d, J = 7.6 Hz, 1H), 7.70 (t, J = 7.6 Hz, 1H), 7.67-7.47 (m, 3H), 4.46 (t, J = 7.2 Hz, 2H), 2.14-1.99 (m, 2H), 0.99 (t, J = 7.2 Hz, 3H).; ^{13}C NMR δ 182.2, 138.5, 138.2, 133.7, 131.6, 131.5, 129.8, 129.2, 128.9, 122.8, 122.3, 115.7, 52.2, 24.2, 11.9.; HRMS for $C_{17}H_{13}ClN_2O$+Na 319.0614; found 319.0615.

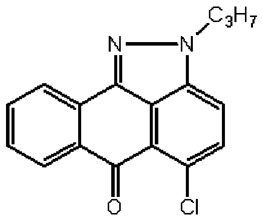

7-chloro-2-propyldibenzo[cd,g]indazol-6(2H)-one (D5): This compound was made by using the same procedure as for D2. The compound is purified by column chromatography (1%

methanol in $CHCl_3$). Yellow color bulge shaped needles suitable for X-ray diffraction were obtained from a mixture of solvents ($CHCl_3$/DCM) at ambient temperatures. X-ray reflections of this molecule were refined in a trigonal space group *R*-3, Z=18 with one molecule in the asymmetric unit; ^{1}H NMR (400, $CDCl_3$) δ 8.19 (d, *J* = 7.6 Hz, 1H), 8.00 (d, *J* = 6.8 Hz, 1H), 7.68 (t, *J* = 7.6 Hz, 1H), 7.64 (dd, *J* = 7.2, 1.2 Hz, 1H), 7.55 (t, *J* = 8.0 Hz, 1H), 7.52 (dd, *J* = 6.8, 1.6 Hz, 1H), 4.49 (t, *J* = 7.2 Hz, 2H), 2.06 (sextet, *J* = 7.2 Hz, 2H), 0.99 (t, *J* = 7.2 Hz, 3H).; ^{13}C NMR (100 MHz, $CDCl_3$): δ 183.2, 139.5, 138.4, 137.7, 135.3, 133.4, 132.9, 129.6, 128.9, 127.5, 123.2, 122.2, 121.3, 115.1, 52.3, 24.2, 11.9.; HRMS for $C_{17}H_{13}ClN_2O$+Na 319.0614; found 319.0615.

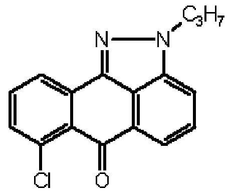

7-chloro-2-methyldibenzo[cd,g]indazol-6(2H)-one (D6): This compound was synthesized by using the same procedure as for D2. Purification is achieved by column chromatography (0.1% methanol in $CHCl_3$). Yellow color needles suitable for X-ray diffraction were obtained from a mixture of solvents ($CHCl_3$/DCM) at ambient temperatures. Single crystal data were solved and refined in a Triclinic space group *P*-1, Z=4 with two molecules in the asymmetric unit (Z'=2);^{1}H NMR (400, $CDCl_3$) δ 8.16 (dd, *J* = 7.2, 2.0 Hz, 1H), 7.98 (dd, *J* = 6.0, 1.6 Hz, 1H), 7.72-7.57 (m, 2H), 7.62-7.46 (m, 2H), 4.24 (s, 3H).; ^{13}C NMR (100 MHz, $CDCl_3$): δ 182.7, 139.4, 138.1, 137.3, 134.7, 132.9, 132.5, 129.1, 128.6, 127.0, 122.8, 121.7, 114.4, 36.5.; HRMS for $C_{15}H_9ClN_2O$+Na calcd 291.0301; found 291.0304.

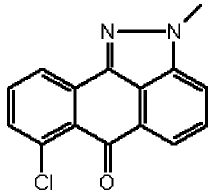

5-chloro-2-methyldibenzo[cd,g]indazol-6(2H)-one (D7): This compound was prepared by using the same procedure as for D4. The compound is purified by column chromatography (1% methanol in $CHCl_3$). Dark yellow color needle shaped single crystals suitable for X-ray diffraction were obtained from a mixture of solvents ($CHCl_3$/DCM) at ambient temperatures. X-ray reflections of this molecule were refined in a monoclinic space group $P2_1/n$, Z=4 with one molecule in the asymmetric unit; ^{1}H NMR (400 MHz, $CDCl_3$) δ 8.45 (d, *J* = 8.0 Hz, 1H), 8.17 (d, *J* = 7.6 Hz, 1H), 7.70 (t, *J* = 7.6 Hz, 1H), 7.62–7.48 (m, 3H), 4.22 (s, 3H).; ^{13}C NMR (100 MHz, $CDCl_3$): δ 181.7, 138.1, 138.0, 133.3, 133.3, 131.3, 130.9, 129.3, 128.9, 128.5, 124.3, 122.3, 121.9, 115.1, 36.5.; HRMS for $C_{15}H_9ClN_2O$+Na calcd 291.0301; found 291.0301.

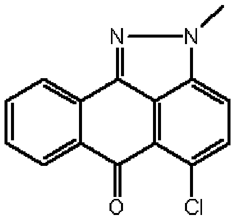

2-allyldibenzo[cd,g]indazol-6(2H)-one (D8): This compound was made by using the same procedure as for D4**.** Compound was purified by column chromatography (1% methanol in $CHCl_3$). ^{1}H NMR (400, $CDCl_3$) δ 8.46 (d, *J* = 8.0 Hz, 1H), 8.24 (d, *J* = 7.6 Hz, 1H), 8.06 (d, *J* = 7.2 Hz, 1H), 7.72 (d, *J* = 8.4 Hz, 2H), 7.65 (t, *J* = 7.6 Hz, 1H), 7.54 (t, *J* = 7.6 Hz, 1H), 6.15 (ddt, *J* = 16.4, 10.8, 5.6 Hz, 1H), 5.33 (d, *J* = 11.2 Hz, 1H), 5.36-5.20 (m, 1H), 5.19 (d, *J* = 5.2 Hz, 2H).; ^{13}C NMR (100 MHz, $CDCl_3$) δ 183.8, 139.1, 133.3, 132.6, 131.8, 129.2, 128.4 (3C), 126.5, 124.0, 122.7, 120.8, 118.5, 115.3, 52.9; HRMS for $C_{17}H_{12}N_2O$+ Na calcd 283.0847; found 283.0852.

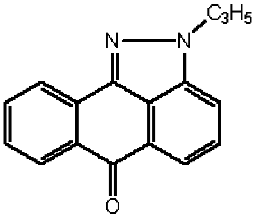

2-(3,3,3-trifluoropropyl)dibenzo[cd,g]indazol-6(2H)-one (D9): The compound was prepared by N-alkylation of 1,9-pyrazoloanthrone with 1,1,1-trifluoro-3-iodopropane. The reaction was carried out in DMSO by keeping temperature at 25°C. ^{1}H NMR (400 MHz, $CDCl_3$) δ 8.45 (d, *J* = 7.6 Hz, 1H), 8.19 (d, *J* = 7.6 Hz, 1H), 8.04 (d, *J* = 4.8 Hz, 1H), 7.78–7.61 (m, 3H), 7.55 (t, *J* = 7.6 Hz, 1H), 4.75 (t, *J* = 7.2 Hz, 2H), 3.02–2.81 (m, 2H); ^{13}C NMR (100 MHz, $CDCl_3$) δ 183.6, 139.9, 139.1, 133.4, 133.3, 131.5, 129.3, 128.8, 128.7, 126.6, 123.6, 122.8, 121.0, 114.3, 42.9, 34.44 (q, *J* = 28.8 Hz).; HRMS (ESI) calcd for $C_{17}H_{11}F_3N_2O$+ Na calcd 339.0721; found 339.0721.

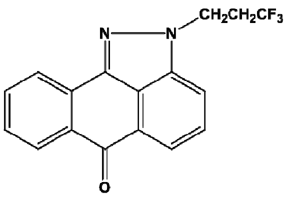

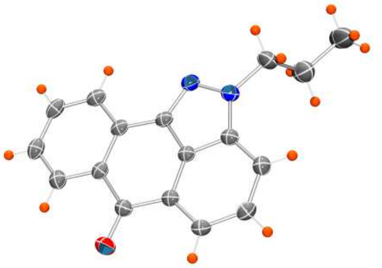	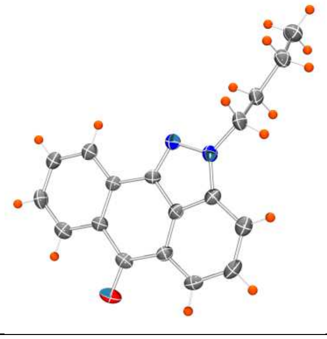
**SPP1**	**SPB1**
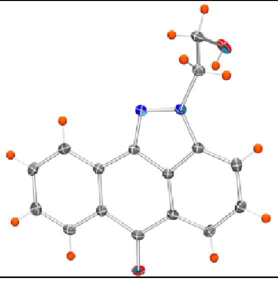	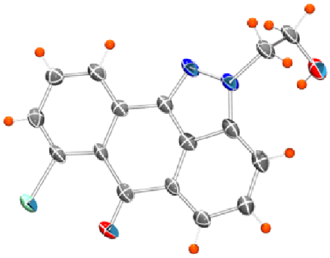
**D1**	**D2**
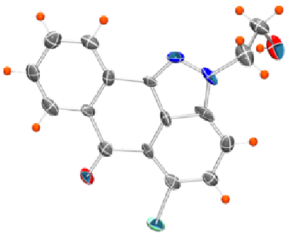	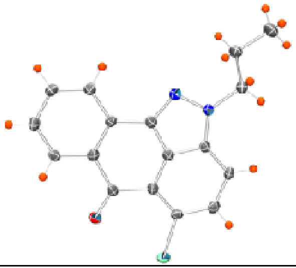
**D3**	**D4**
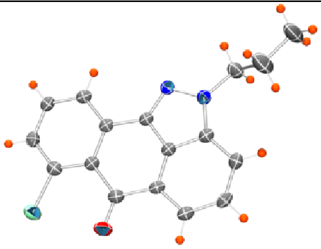	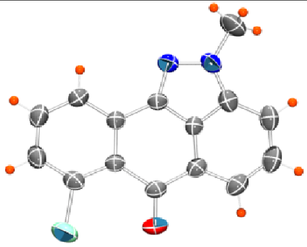
**D5**	**D6**
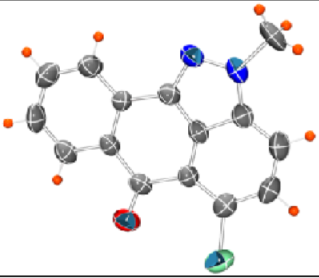	
**D7**	

Figure S3 ORTEPs of anthrapyrazolone analogues with displacement ellipsoids at 50% probability level.

Table S1 Crystallographic parameters of synthesized anthrapyrazolone analogues.

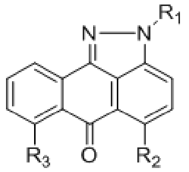

Compound	R_1	R_2	R_3	Cell parameters and CCDC Nos.
SPP1	C_3H_7	H	H	*R*-3; *a=27.789(5), b=27.789(5), c=8.948(3);* *γ=120; Volume=5984.5(5),* **CCDC No: 990060**
SPB1	C_4H_9	H	H	$P2_12_12_1$; *a=4.909(5), b=14.694(2), c=19.032(2);* *Volume=1372.9(3),* **CCDC No: 990059**
D1	C_2H_5OH	H	H	*P*-1; *a=7.201(8), b=8.025(1), c=11.013(1);* *α=85.61(1), β=72.65(1), γ=86.95(1)* *Volume=605.4(1),* **CCDC No: 1018860**
D2	C_2H_5OH	H	Cl	$P2_1/c$; *a=14.207(8)(8), b=6.954(4), c=25.685(1);* *β=90.71(5); Volume=2537.7(2),* **CCDC No: 1018861**
D3	C_2H_5OH	Cl	H	$Pca2_1$; *a=7.690(5), b=12.227(7), c=27.890(2);* *Volume=2622.7(3),* **CCDC No: 1018862**
D4	C_3H_7	Cl	H	$P2_12_12_1$; *a=3.985(2), b=16.297(8), c=20.895(8);* *Volume=1357.1(1),* **CCDC No: 1018863**
D5	C_3H_7	H	Cl	*R*-3; *a=30.094(1), b=30.094(1), c=7.795(4); γ=120;* *Volume=6114.0(7),* **CCDC No: 1018864**
D6	CH_3	Cl	H	*P*-1; *a=8.132(4), b=11.836(6), c=12.383(6);* *α=88.47(4), β=84.41(9), γ=87.47(4)* *Volume=1185.0(1),* **CCDC No: 1018859**
D7	CH_3	H	Cl	$P2_1/n$; *a=3.865(1)(8), b=15.774(4), c=19.287(6);* *β=94.97(3); Volume=1171.7(6),* **CCDC No: 1018865**
D8	C_3H_5	H	H	Single crystal could not be obtained
D9	$C_3H_4F_3$	H	H	-do-

Docking: The three-dimensional (3D) structures of all inhibitors were modeled and minimized using the PRODRG server[10] and single crystal structures. AutoDock (version 4.2)[11] was used for the ligand-protein docking. The Lamarckian Genetic Algorithm was used with a population of 200 dockings. The docking output was analysed using Pymol and Ligplot.[12] Hydrogen bonds were determined using the in-built HBPLUS[13] module in Ligplot with hydrogen bonding parameters (D···A distance≤3.35 Å, H···A≤2.7 Å).

Table S2 Details showing the various interactions of anthrapyrazolone (1,9-pyrazoloanthrone or SP600125) and its analogues with 1PMV(JNK3).

	**Compounds**	**Hydrogen bonds**	**Distance between donor and acceptor (Å)**	**Halogen interactions**	**Distance(Å)**	**Binding Energy (Kcals)**
	SP600125	Glu(147) O···N24	2.72	NA	NA	-8.05
	SPB1	Met(149) N···O	2.91	NA	NA	-8.12
	SPP1	Met(149) N···O	2.62	NA	NA	-7.88
	D1	Met(149) N···OAA<br>Asn(152) ND2···OAB	2.55<br>3.01	NA	NA	-8.23
**Protein 1PMV**	D2	Met(149) N···O<br>Lys(93) NZ···O15	2.91<br>2.86	Met(149) O···Cl2<br>Asp(150) O···Cl2	3.2<br>3.6	-8.40
	D3	Met(149) N···O<br>Asn(152) ND2···O0A	2.84<br>2.98	Met(149) O···Cl<br>Asp(150) O···Cl	3.2<br>3.7	-8.42
	D4	Met(149) N···O	2.87	Met(149) O···Cl1<br>Asp(150) O···Cl1	3.2<br>3.7	-8.40
	D5	Met(149) N···O	2.92	Met(149) O···Cl<br>Asp(150) O···Cl	3.3<br>3.9	-8.45
	D6	Met(149) N···O	2.97	Met(149) O···Cl2<br>Asp(150) O···Cl2	3.3<br>3.6	-8.07
	D7	Met(149) N···O1	2.86	Met(149) O···Cl1<br>Asp(150) O···Cl1	3.2<br>3.7	-8.05
	D8	Met(149) N···OAB	2.61	NA	NA	-7.97
	D9	Met(149) N···OAA	2.68	Ser(72) N···FAD<br>Ser(72) O···FAD<br>Ser(72) N···FAC	2.92<br>3.44<br>3.28	-7.89

Table S3 Details showing the various interactions of anthrapyrazolone (1,9-pyrazoloanthrone or SP600125) and its analogues with 3E70(JNK2)

	**Small molecule**	**Hydrogen bonds**	**Distance between donor and acceptor (Å)**	**Halogen interactions**	**Distance(Å)**	**Binding energy**
	SP600125	No H bond	NA	NA	NA	-8.57
	SPB1	Leu(206) O···N3	3.13	NA	NA	-8.44
	SPP1	No H bond	NA	NA	NA	-8.44
	D1	Lys(93) NZ···OAB	2.80	NA	NA	-8.43
	D2	Leu(206) O···N5<br>Arg(107) NH2···O15	2.97<br>3.16	Ala(91) O···Cl2<br>Lys(93) N···Cl2<br>Leu(144) O···Cl2<br>Met(146) N···Cl2	2.94<br>3.43<br>3.08<br>3.78	-8.79
	D3	Leu(206) O···N11<br>Lys(93) NZ···O0A	3.25<br>2.84	Ala(91) O···Cl<br>Leu(144) O···Cl<br>Val(145) N···Cl<br>Met(146) N···Cl	3.07<br>3.11<br>3.76<br>3.32	-8.84
	D4	Lys(93) NZ···O	2.84	Lys(93) NZ···Cl1	3.29	-8.29
**Protein 3E70**	D5	Leu(206) O···N4	3.07	Ala(91) O···Cl<br>Lys(93) N···Cl<br>Leu(144) O···Cl	2.96<br>3.66<br>3.08	-8.65

				Met(146) N···CL	3.57	
	D6	Leu(206) O···N2	3.05	Ala(91) O···Cl2<br>Lys(93) N···Cl2<br>Leu(144) O···Cl2<br>Met(146) N···Cl2	2.94<br>3.47<br>3.07<br>3.73	-8.47
	D7	No H bond	NA	Ala(91) O···Cl1<br>Leu(144) O···Cl1<br>Met(146) N···Cl1	3.00<br>2.96<br>3.22	-8.15
	D8	No H bond	NA	NA	NA	-8.67
	D9	Met(111) N···OAA	2.66	Lys(93) NZ···FAB<br>Lys(93) NZ···FAD<br>Asn(194)<br>OD1···FAD	2.99<br>3.60<br>3.13	-7.76

Table S4 Details showing the various interactions of anthrapyrazolone (1,9-pyrazoloanthrone or SP600125) and its analogues with 2NO3(JNK1)

	Small molecule	Hydrogen bonds	Distance between donor and acceptor (Å)	Halogen interactions	Distance(Å)	Binding energy
**Protein 2NO3**	SP600125	Ser(155) O···N23	3.13	NA	NA	-6.26
	SPB1	Asn(114) ND2···O	2.81	NA	NA	-6.60
	SPP1	Asn(114) ND2···O	2.95	NA	NA	-6.43
	D1	Asn(114) ND2···OAA<br>Met(111) N···OAB	2.91<br>2.93	NA	NA	-6.96
	D2	Asn(114) ND2···O1<br>Met(111) N···O15	3.03<br>2.86	Ser(155) O···Cl2	3.3	-6.66
	D3	Asn(114) ND2···O<br>Met(111) N···O0A	3.11<br>2.88	Asn(114) N···Cl	3.12	-6.43
	D4	Asn(114) ND2···O	3.08	Asn(114) N···Cl	3.11	-6.81
	D5	Asn(114) ND2···O	2.93	Asn(114) HD2···Cl<br>Ser(155) O···Cl	3.37<br>3.44	-6.93
	D6	Asn(114) ND2···O2	3.15	Asn(114) HD2···Cl<br>Ser(155) O···Cl	3.35<br>3.12	-6.36
	D7	No H bond	NA	Asn(114) N···Cl1	3.46	-6.37
	D8	Asn(114) ND2···OAB	2.91	NA	NA	-6.67
	D9	Asn(114) ND2···OAA	3.10	Glu(109) O···FAC<br>Glu(109) O···FAB<br>Leu(110) N···FAB<br>Met(111) HN···FAD	2.46<br>2.87<br>3.14<br>3.46	-6.3

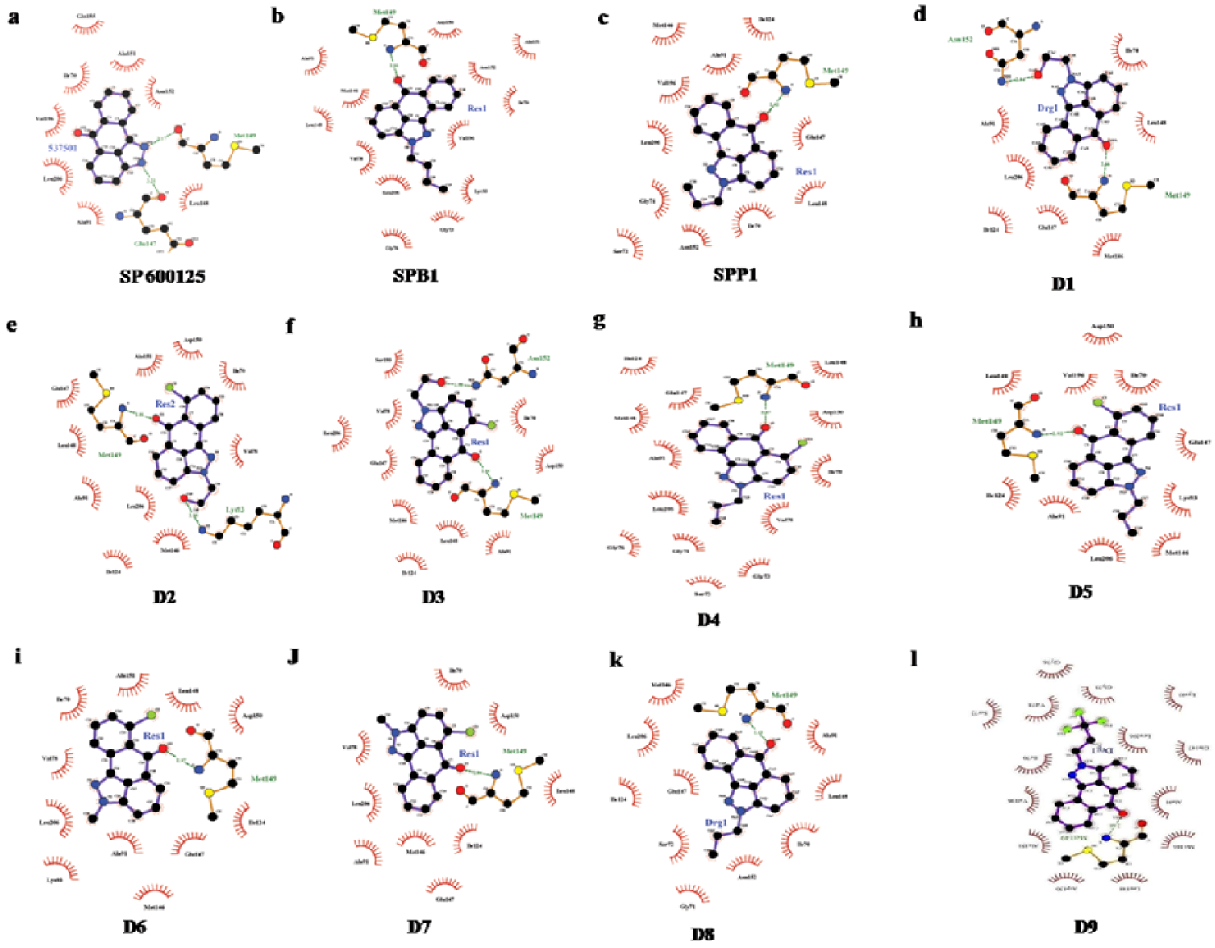

Figure S4 Lig plots for JNK3 (1PMV) with anthrapyrazolone (SP600125) and its analogues.

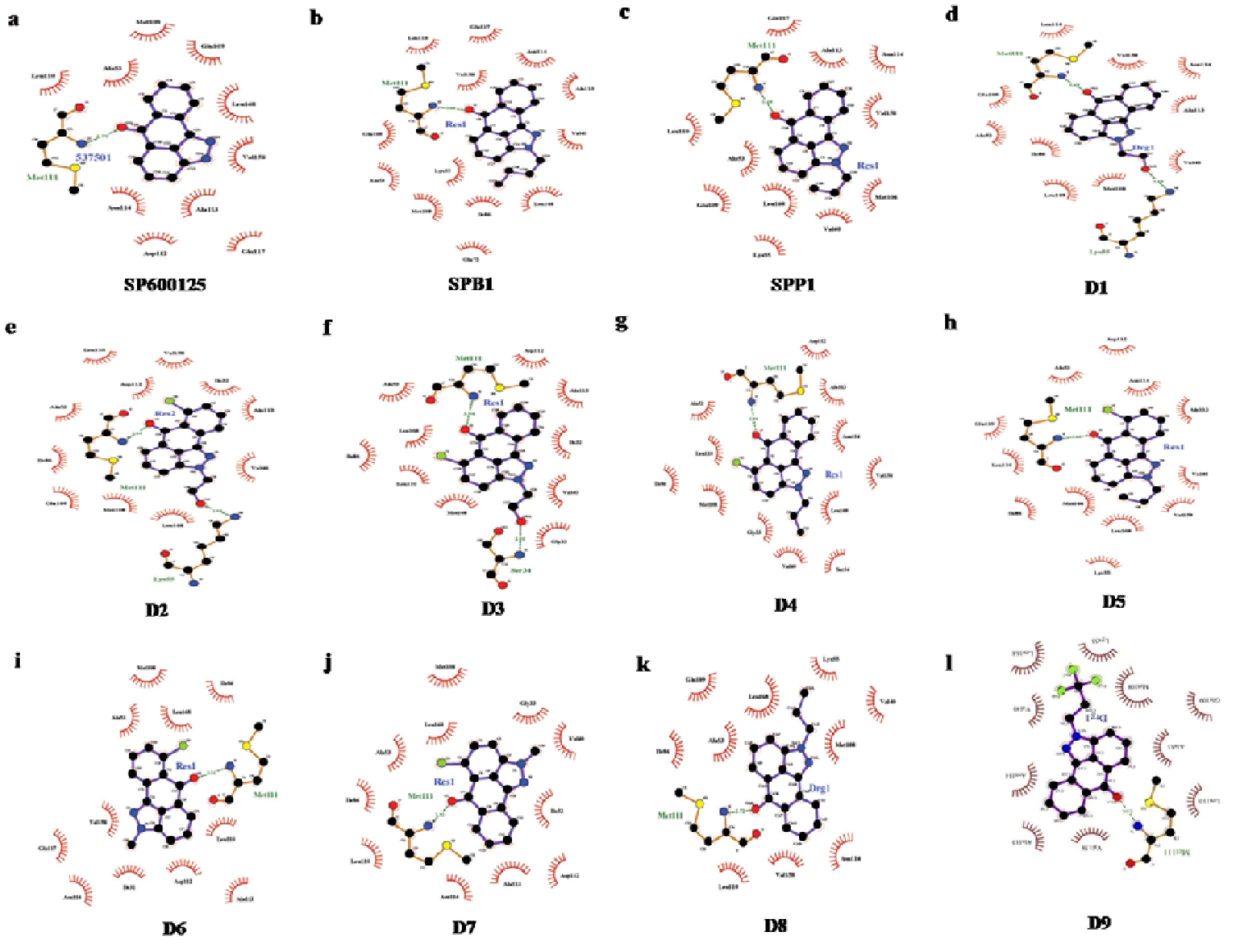

Figure S5 Lig plots for JNK2 (3E7O) with anthrapyrazolone (SP600125) and its analogues.

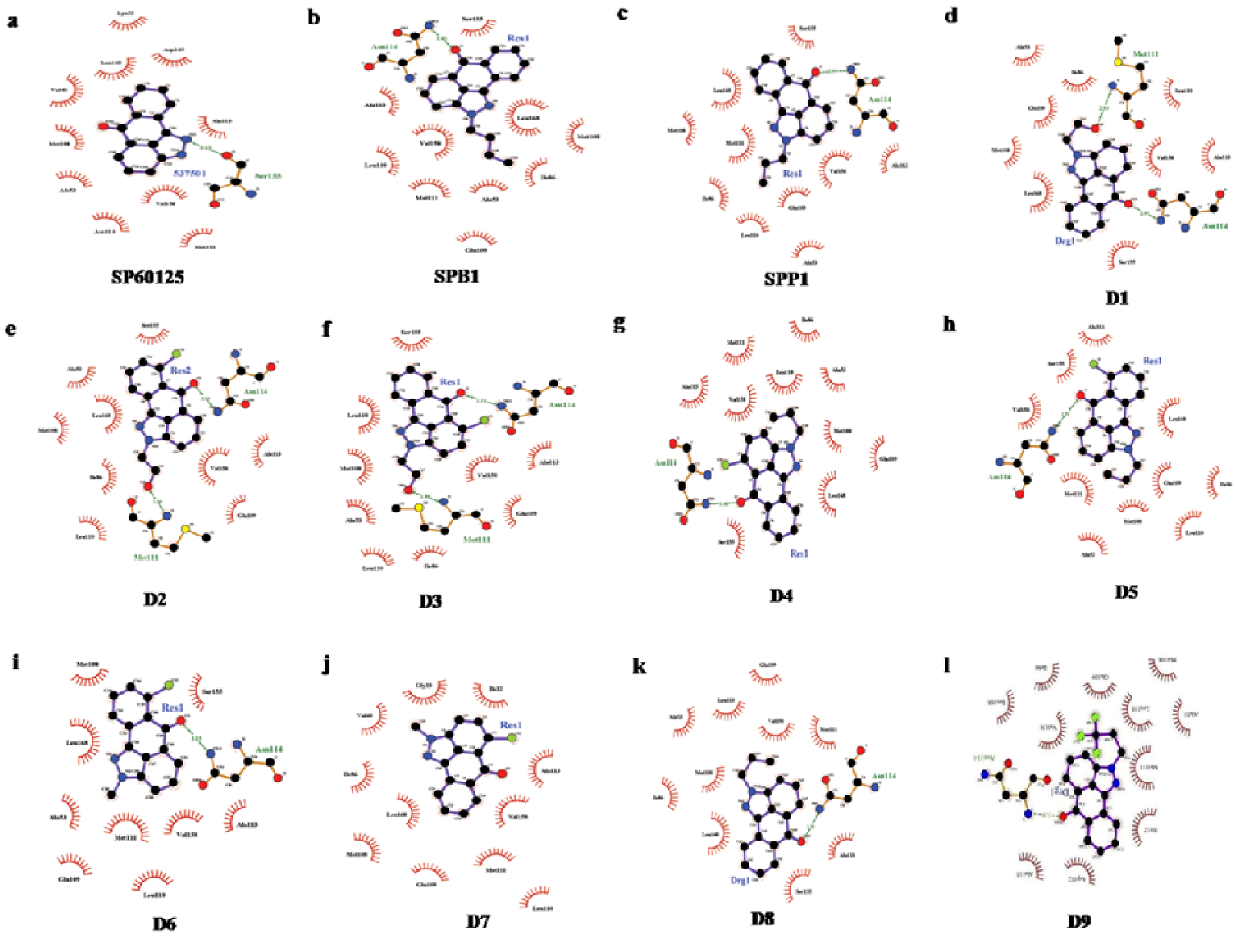

Figure S6 Lig plots for JNK1(2NO3) with anthrapyrazolone (SP600125) and its analogues.

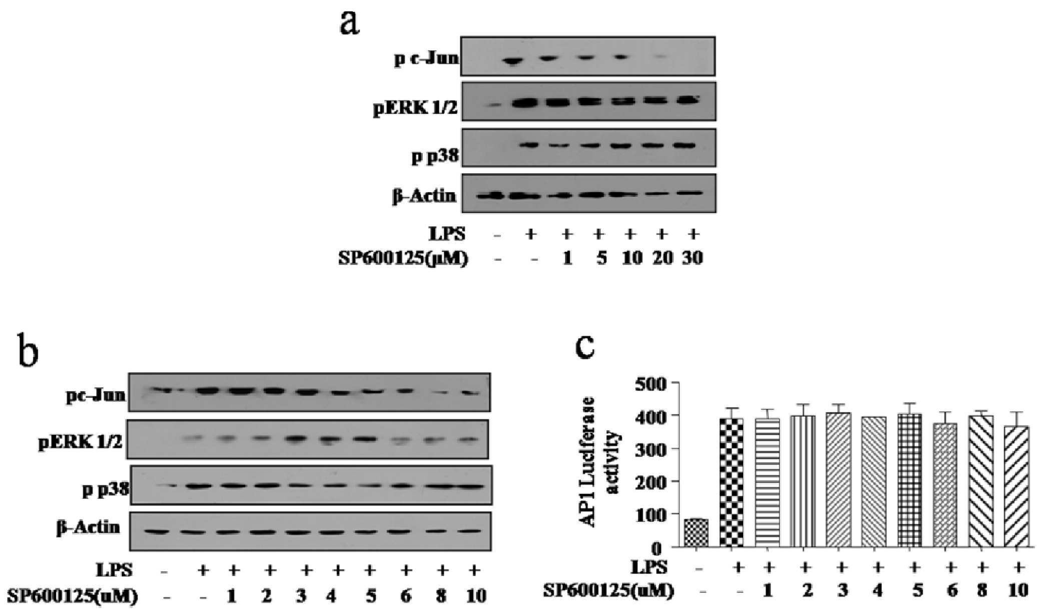

Figure S7 (a) SP600125 (anthrapyrazolone) decreases the phosphorylation of c-Jun at varied concentrations. **(b)** phosphorylaton of c-Jun is inhibited by SP600125 only at concentration greater than 8-10uM. **(c)** SP600125 could not block the transcriptional activity at lower concentration tested as analysed by AP1 luciferase activity.

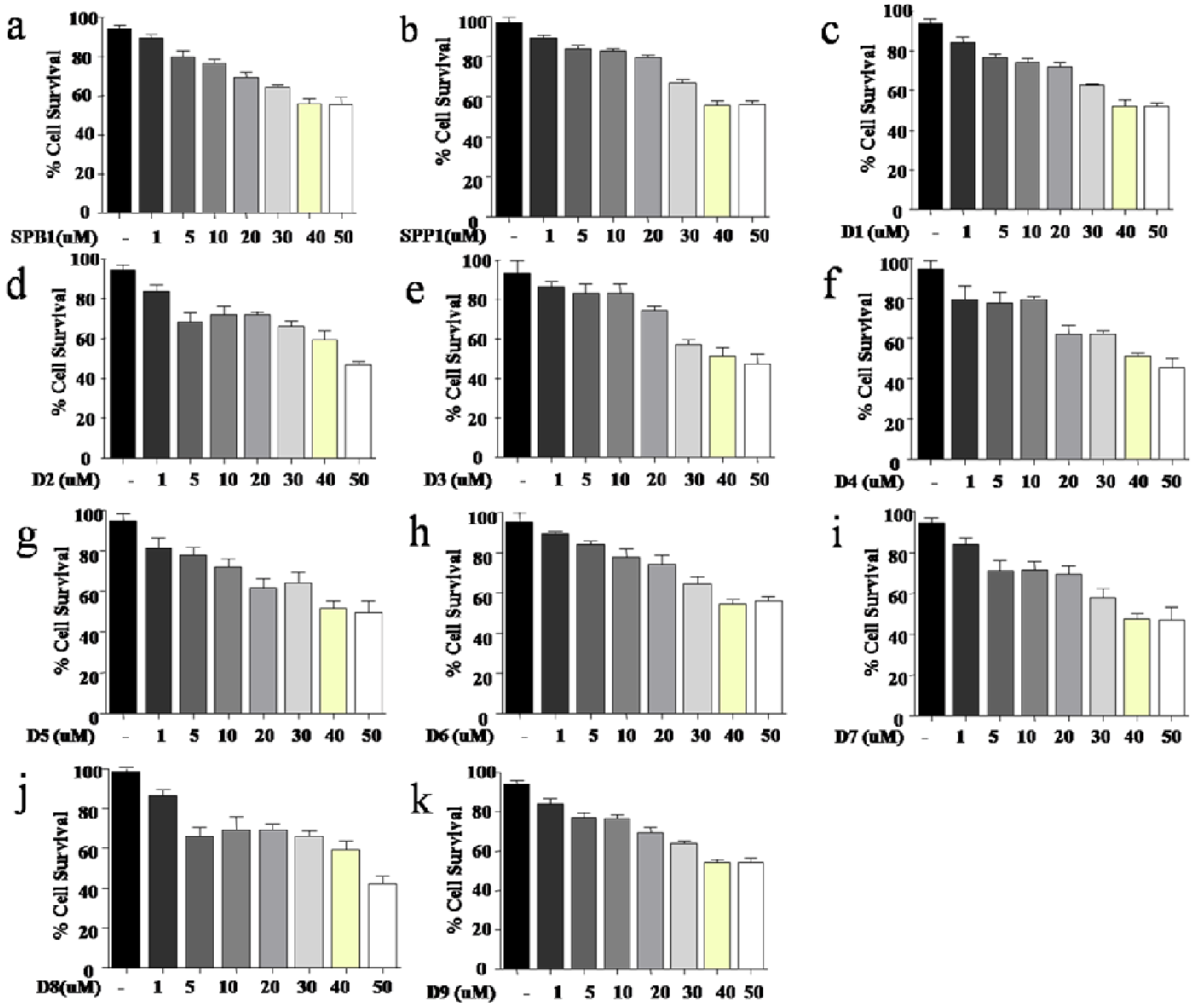

Figure S8 Cell viability analyzed by MTT assay for the inhibitors tested. Viability of macrophages treated with all molecules at varied concentrations tested with **(a)** SPB1, **(b)** SPP1, **(c)** D1, **(d)** D2, **(e)** D3, **(f)** D4, **(g)** D5, **(h)** D6, **(i)** D7, **(j)** D8 and (k) D9 upon treatment for 12 hour followed by which MTT assay was carried out.

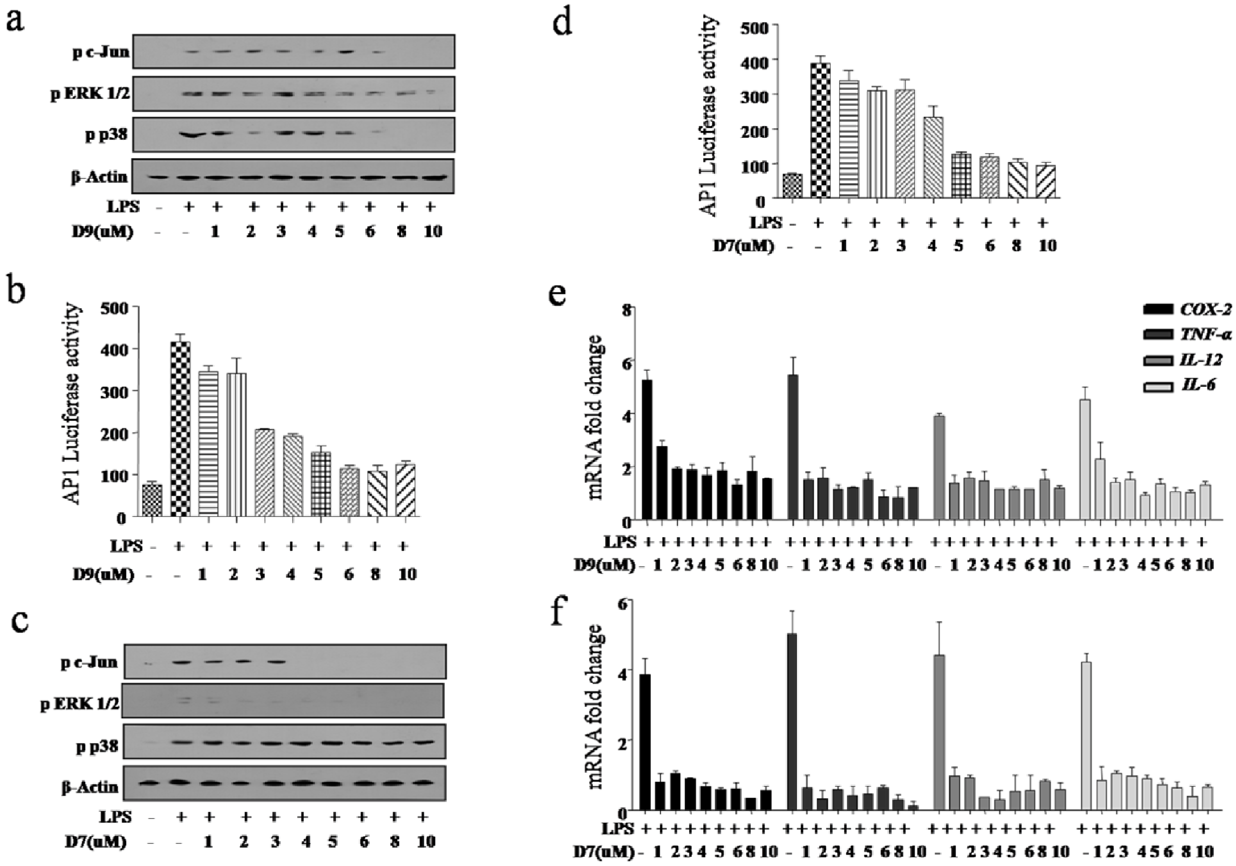

Figure S9 Analogues of anthrapyrazolone inhibit JNK and other MAPkinases in concentration dependent manner. (**a, b**) Inhibitory effect of D9 over phosphorylation of c-Jun at lower concentrations analyzed by western blot and AP1 luciferase activity upon stimulation with LPS for 1hour and 12 hour respectively. (**c, d**) Inhibitory effect of D7 over phosphorylation of c-Jun at lower concentrations analyzed by western blot and AP1 luciferase activity upon stimulation with LPS for 1hour and 12 hour respectively. (**e, f**) Real Time PCR analysis of COX-2, TNF-α, IL-12 and IL-6 in macrophages upon treatment with D9 and D7 at lower concentrations in presence of LPS (100ng/ml) respectively.

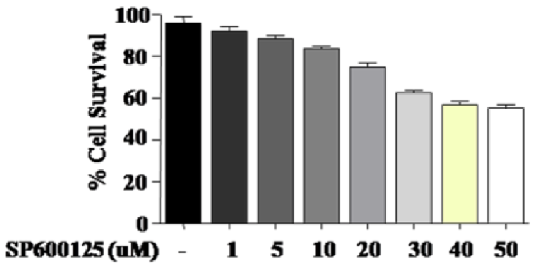

Cell viability assay in the presence of SP600125 (anthrapyrazolone) with varied concentrations analyzed after 12 hour incubation.

^{1}H and ^{13}C NMR of anthrapyrazolone analogues

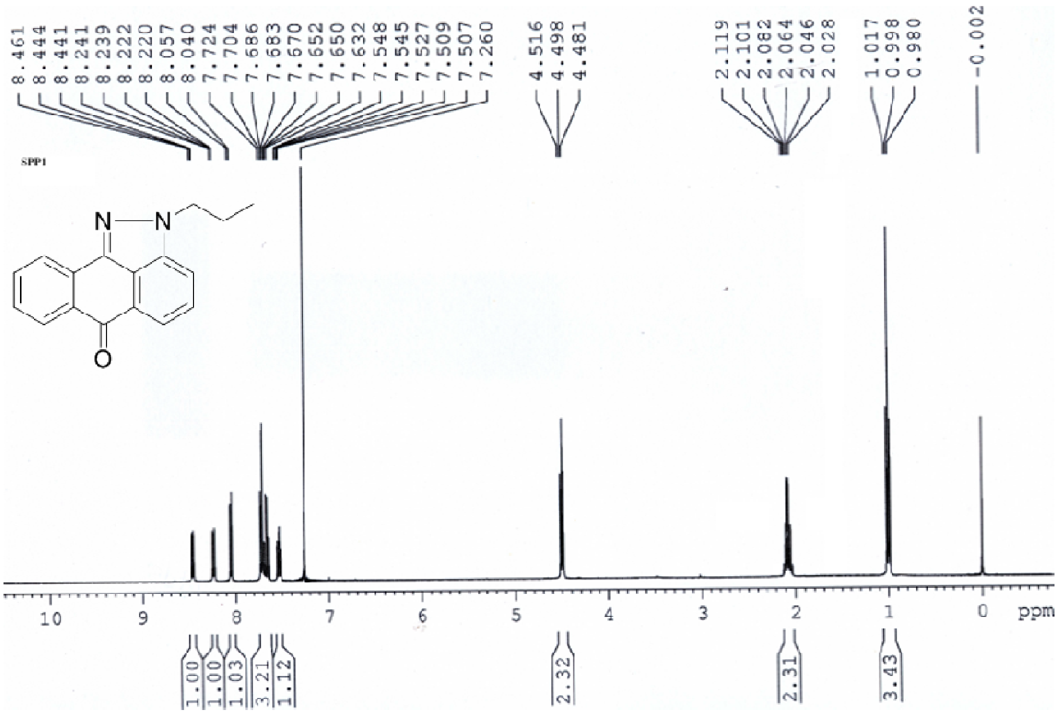

^{1}H NMR spectra of **SPP1**

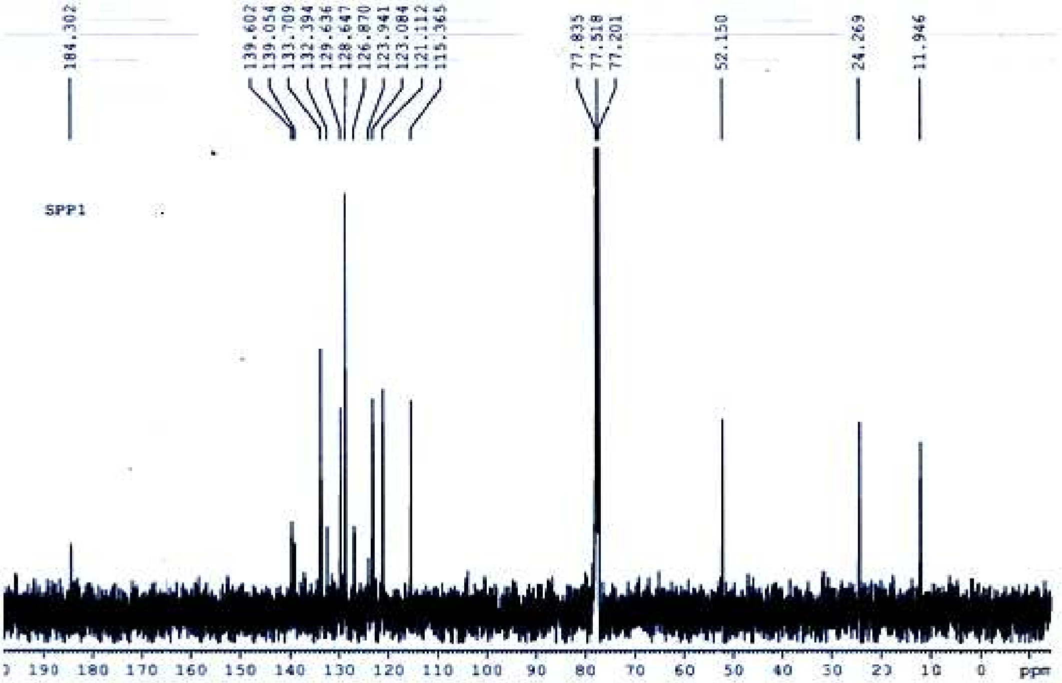

^{13}C NMR spectra of **SPP1**

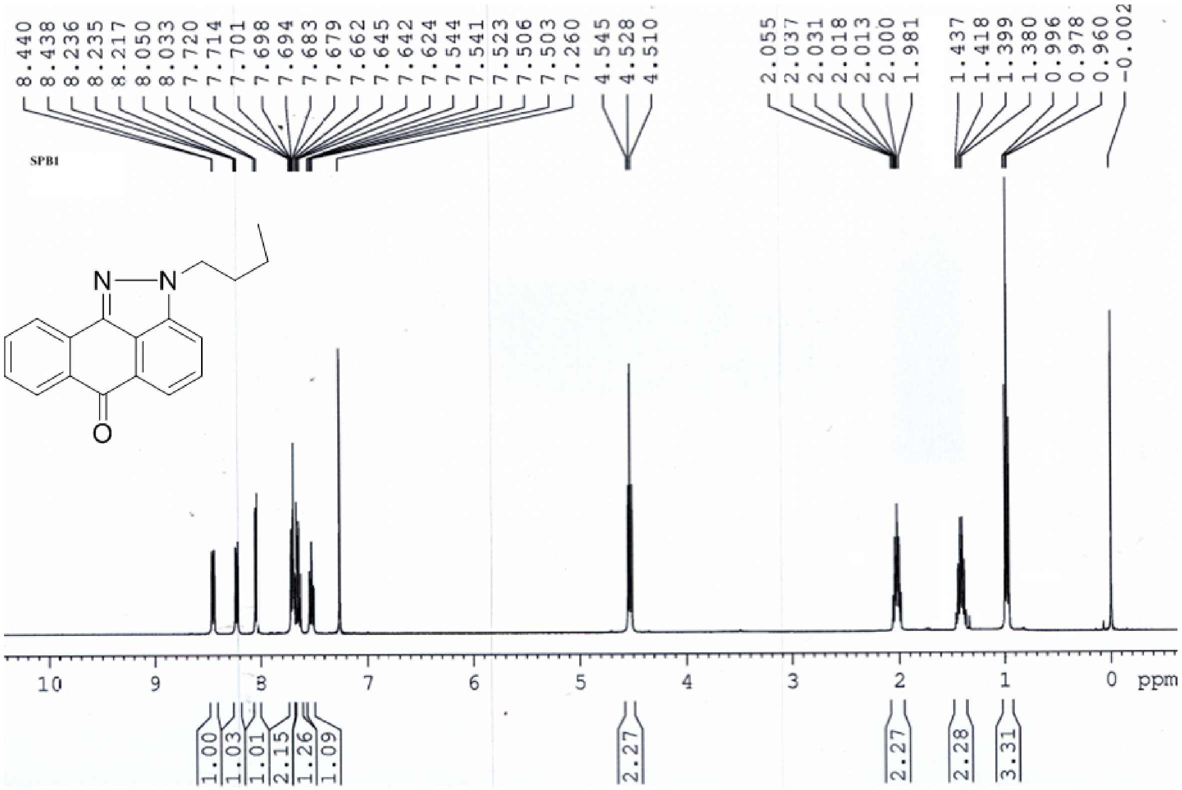

^{1}H NMR spectra of **SPB1**

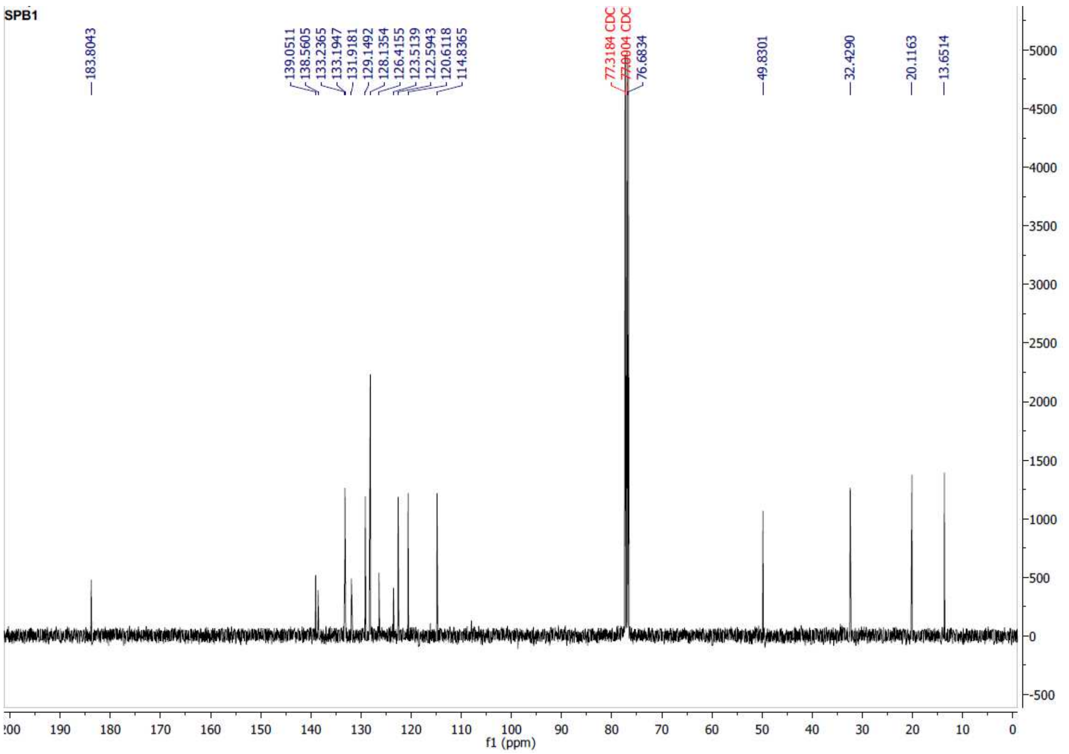

^{13}C NMR spectra of **SPB1**

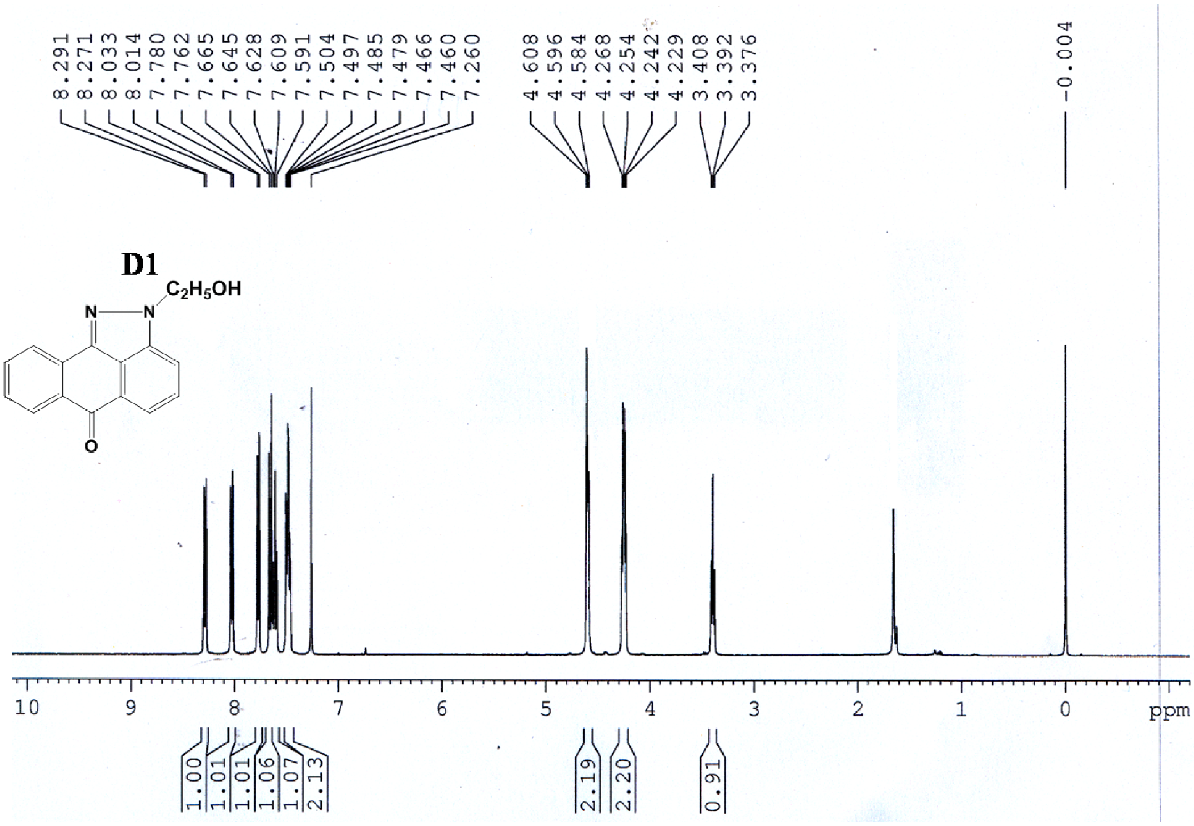

^{1}H NMR spectra of **D1**

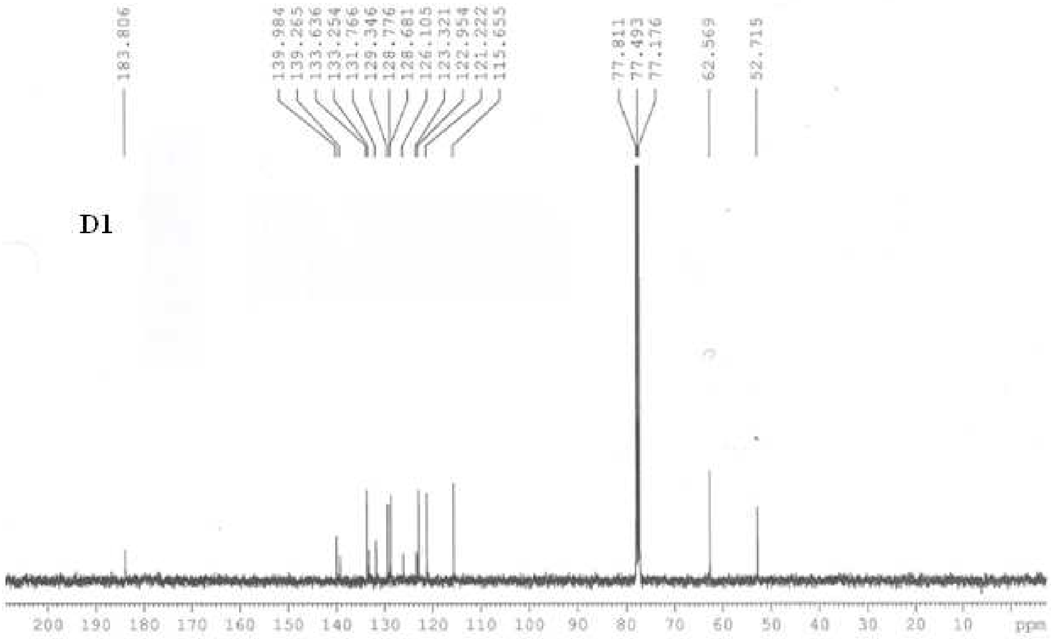

^{13}C NMR spectra of **D1**

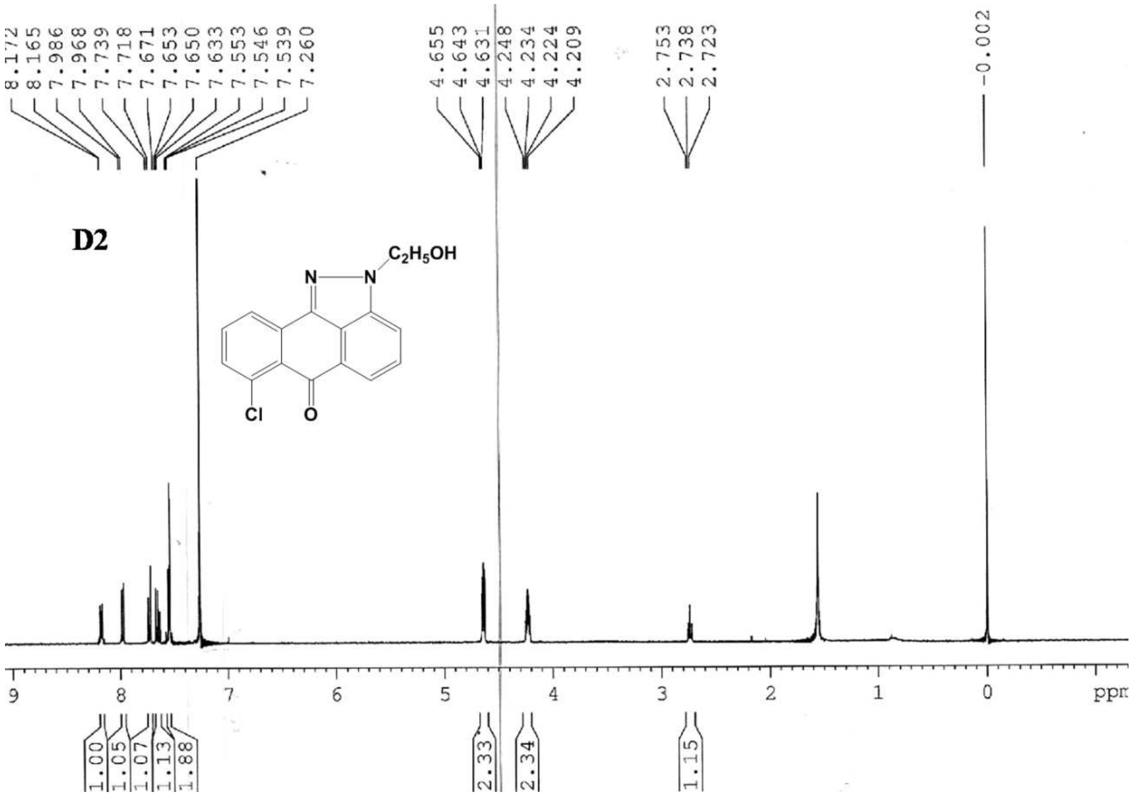

^{1}H NMR spectra of **D2**

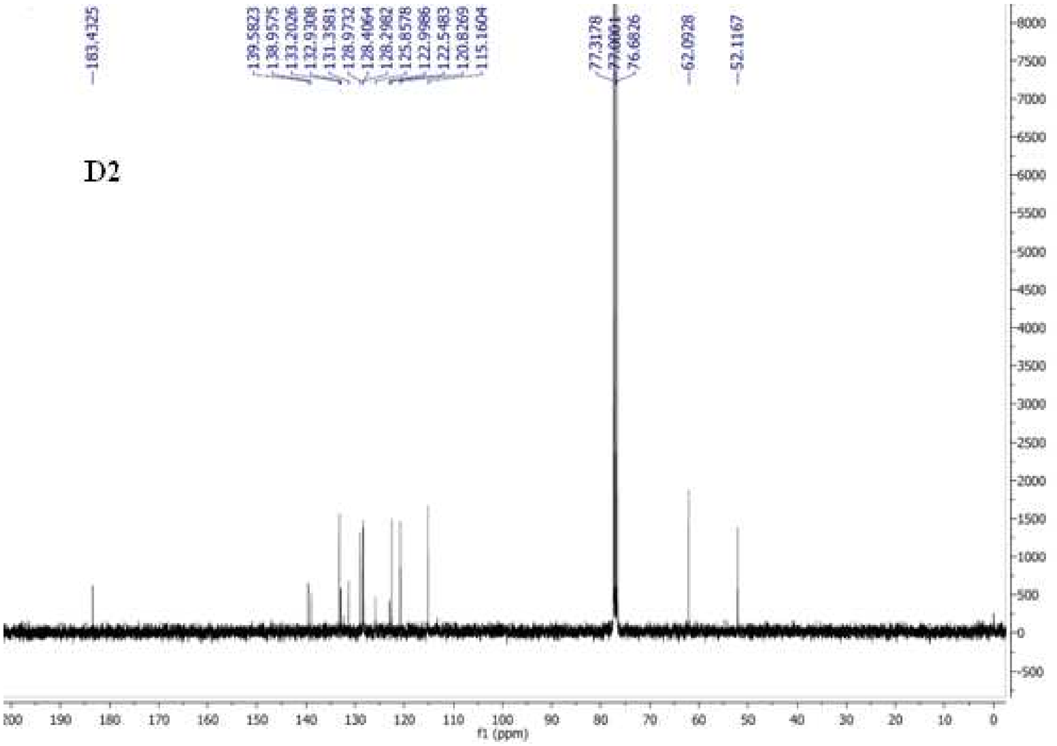

^{13}C NMR spectra of **D2**

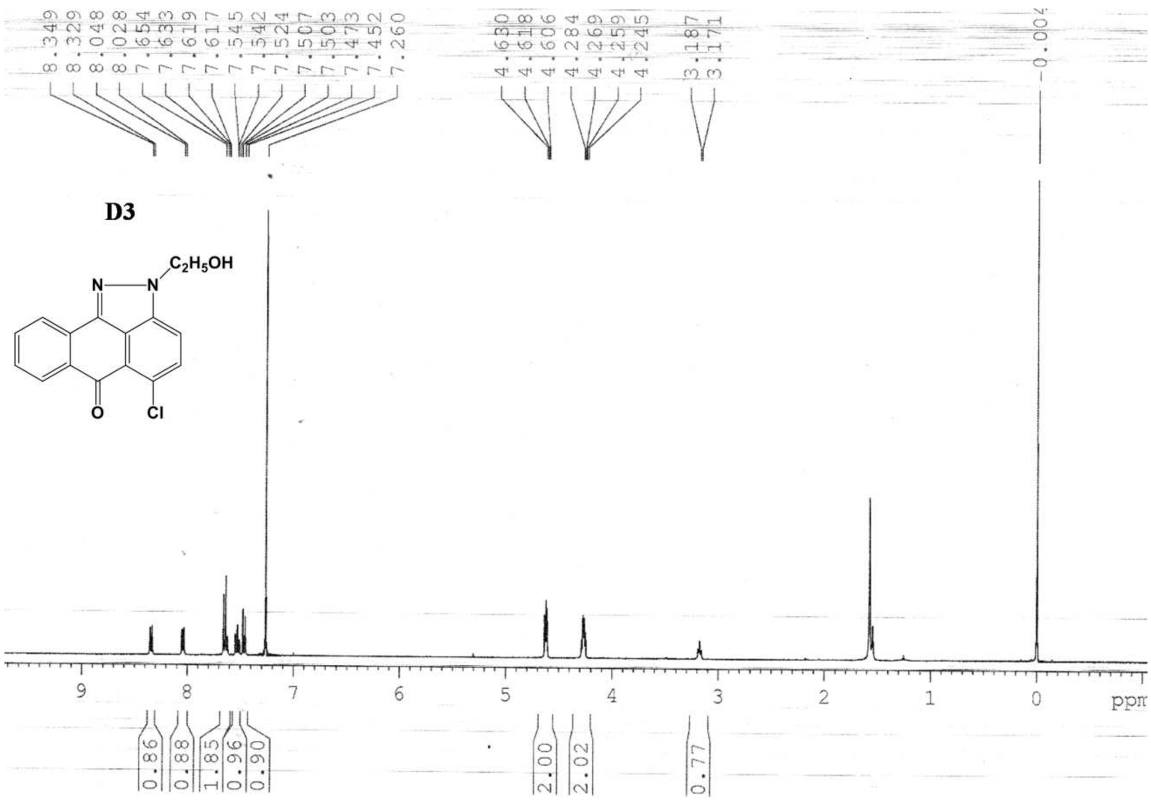

^{1}H NMR spectra of **D3**

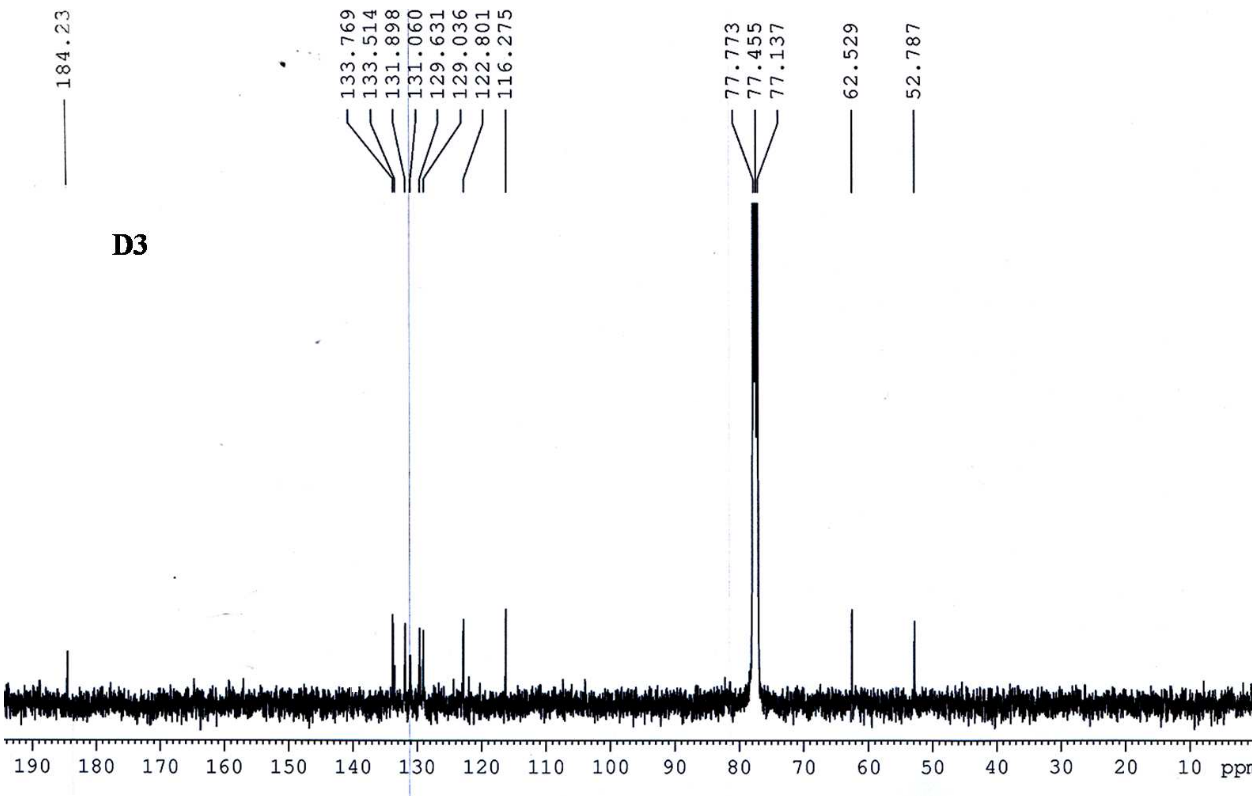

^{13}C NMR spectra of **D3**

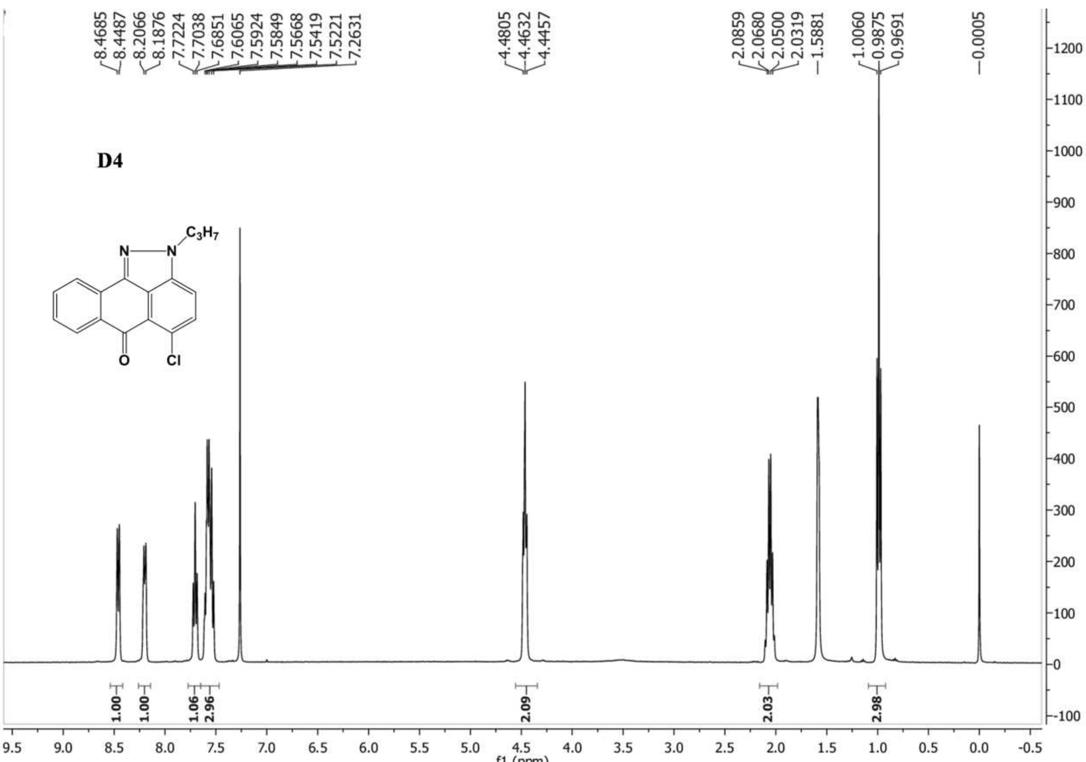

^{1}H NMR spectra of **D4**

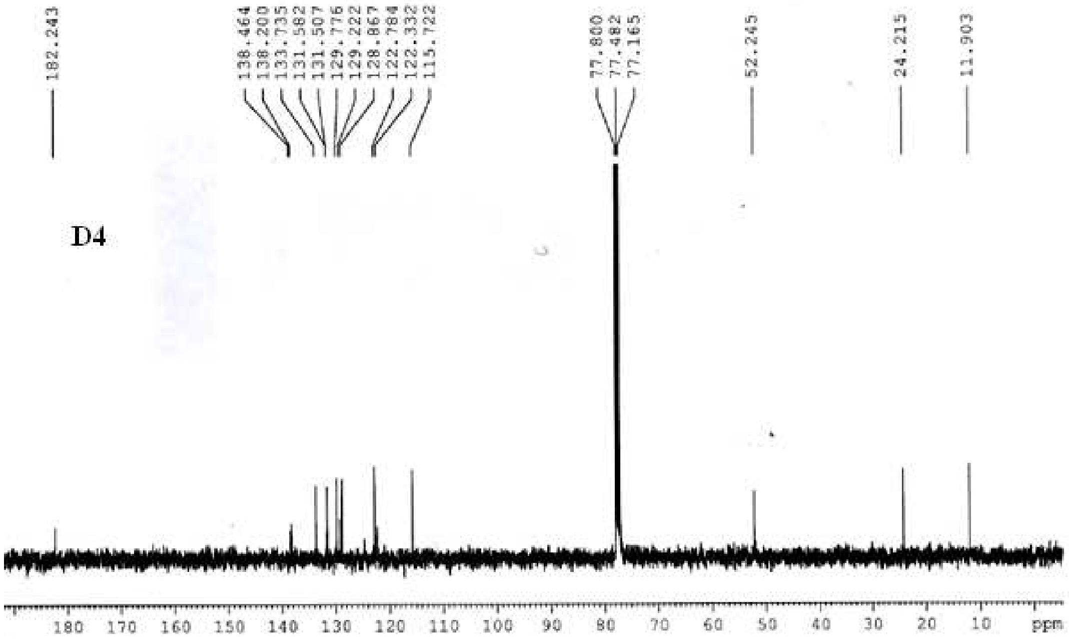

^{13}C NMR spectra of D4

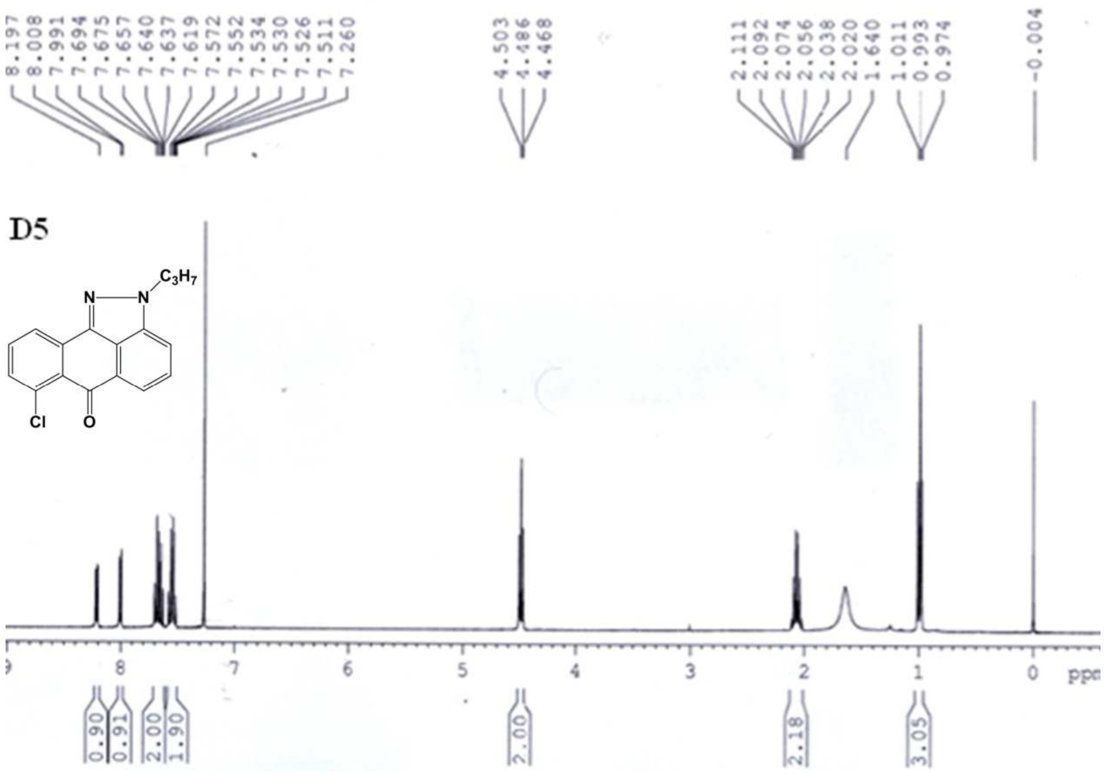

^{1}H NMR spectra of **D5**

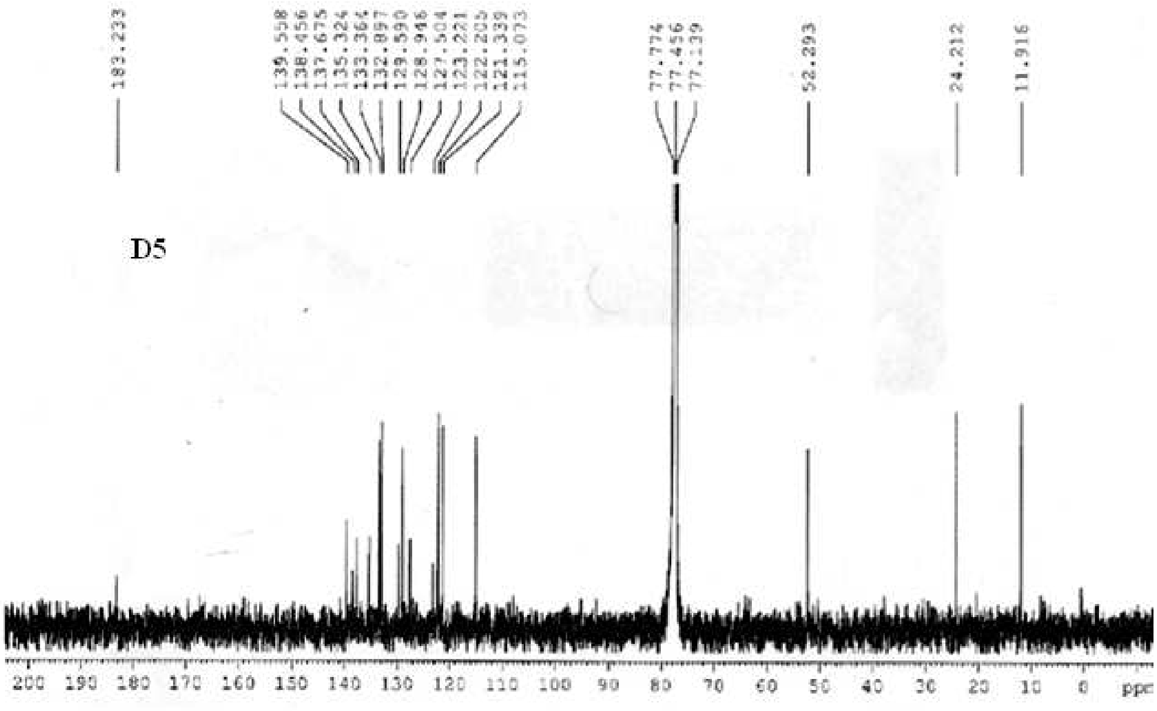

^{13}C NMR spectra of **D5**

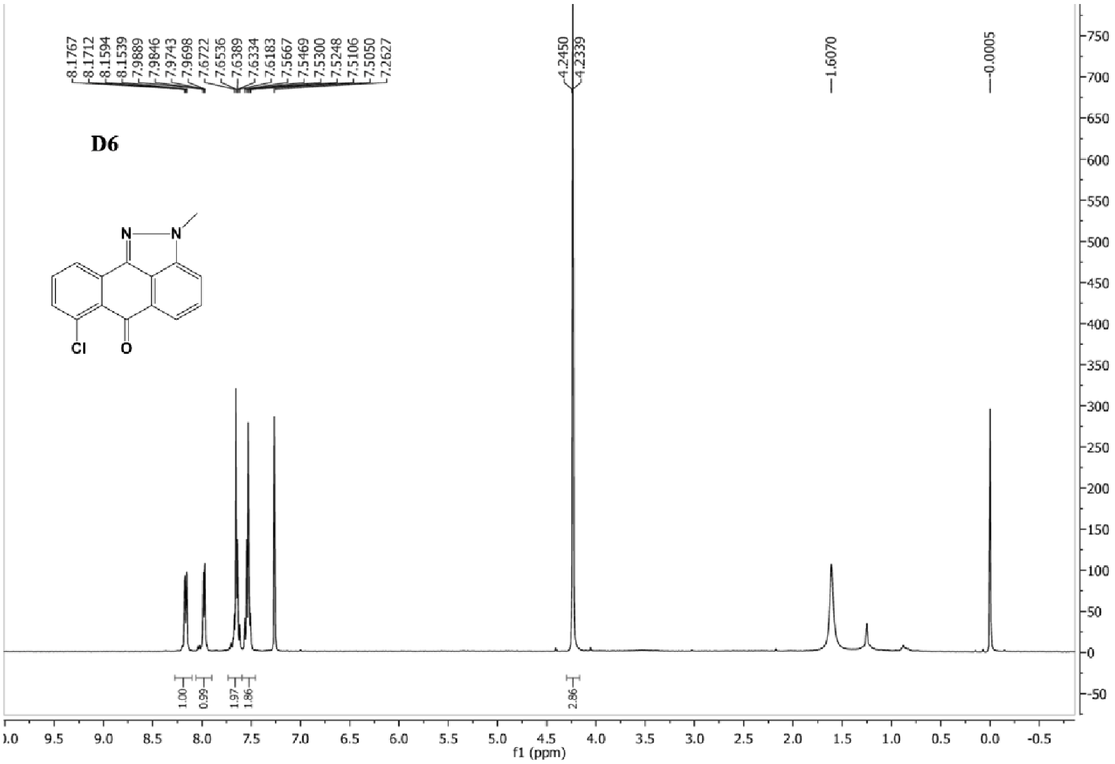

^{1}H NMR spectra of **D6**

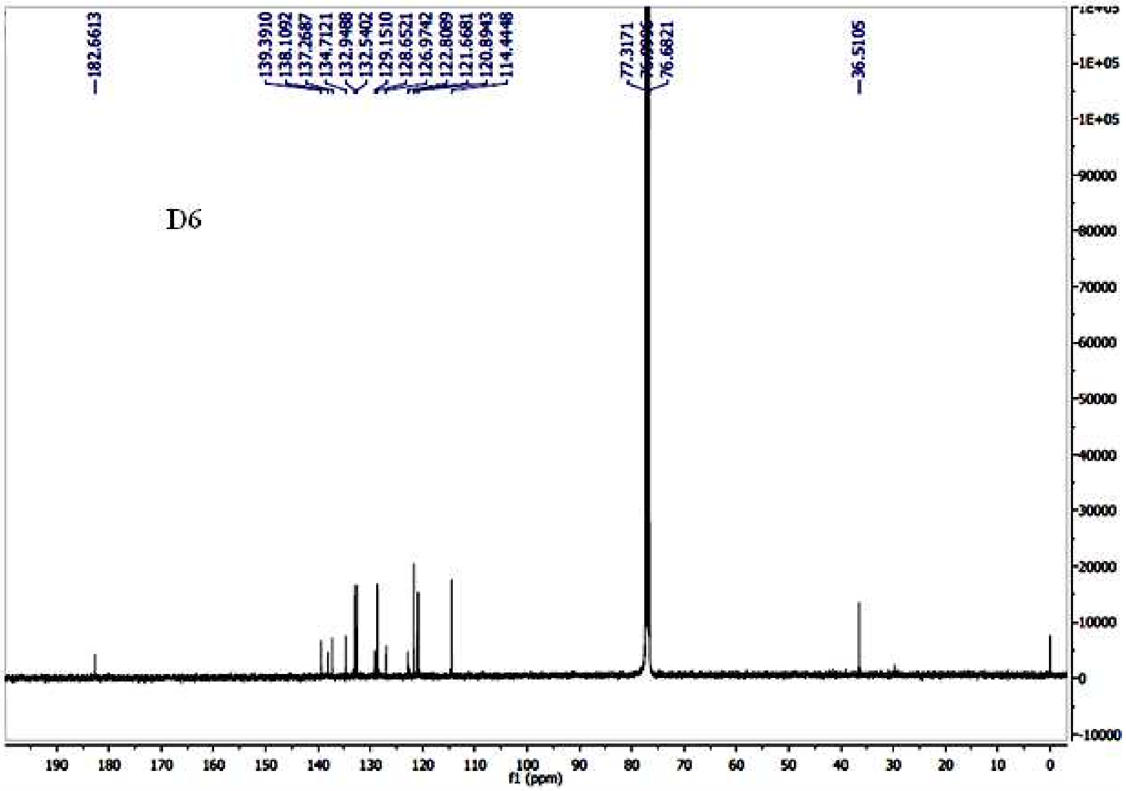

^{13}C NMR spectra of **D6**

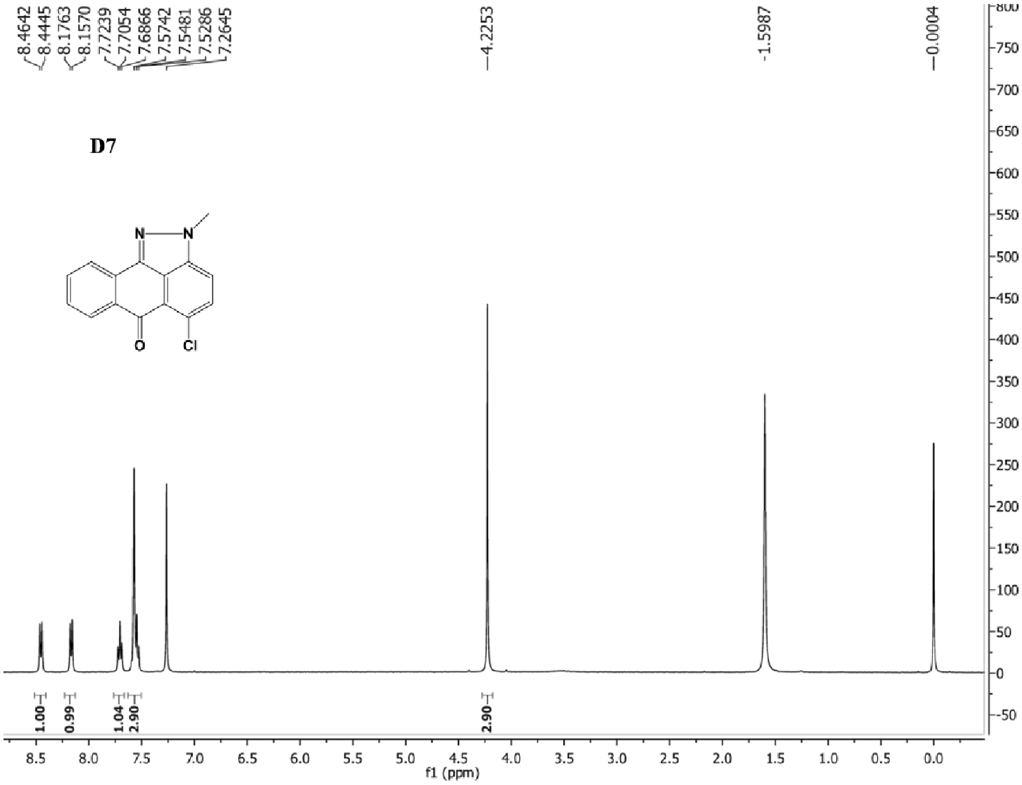

^{1}H NMR spectra of **D7**

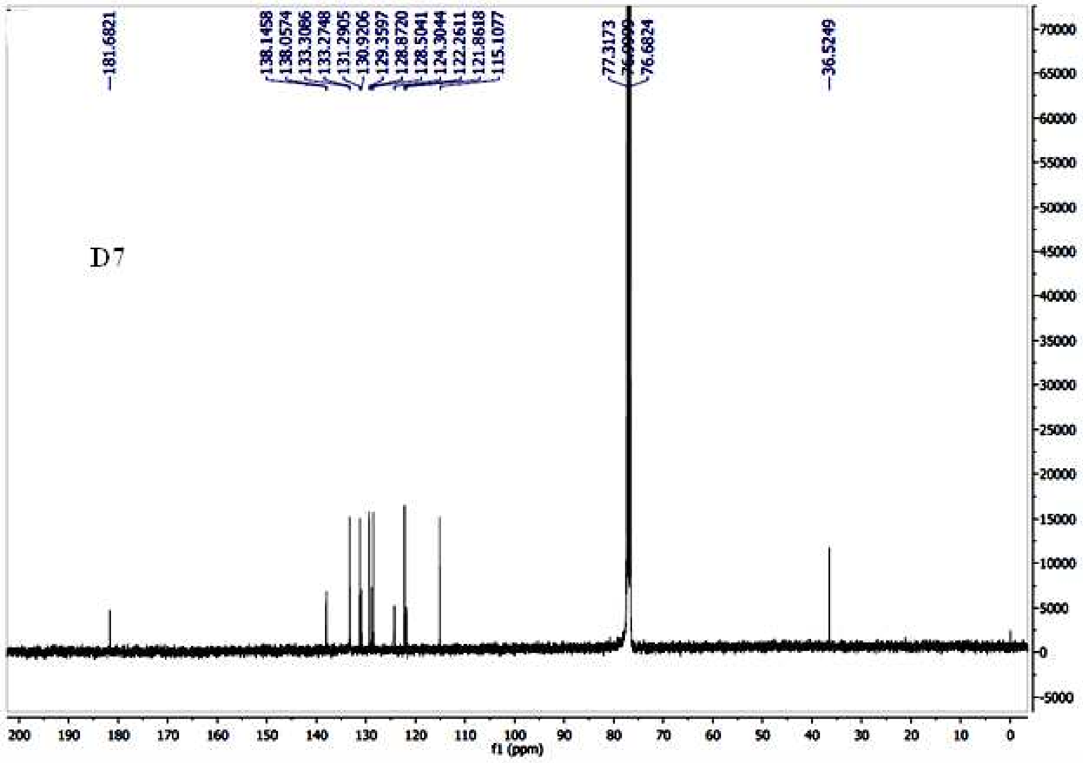

^{13}C NMR spectra of **D7**

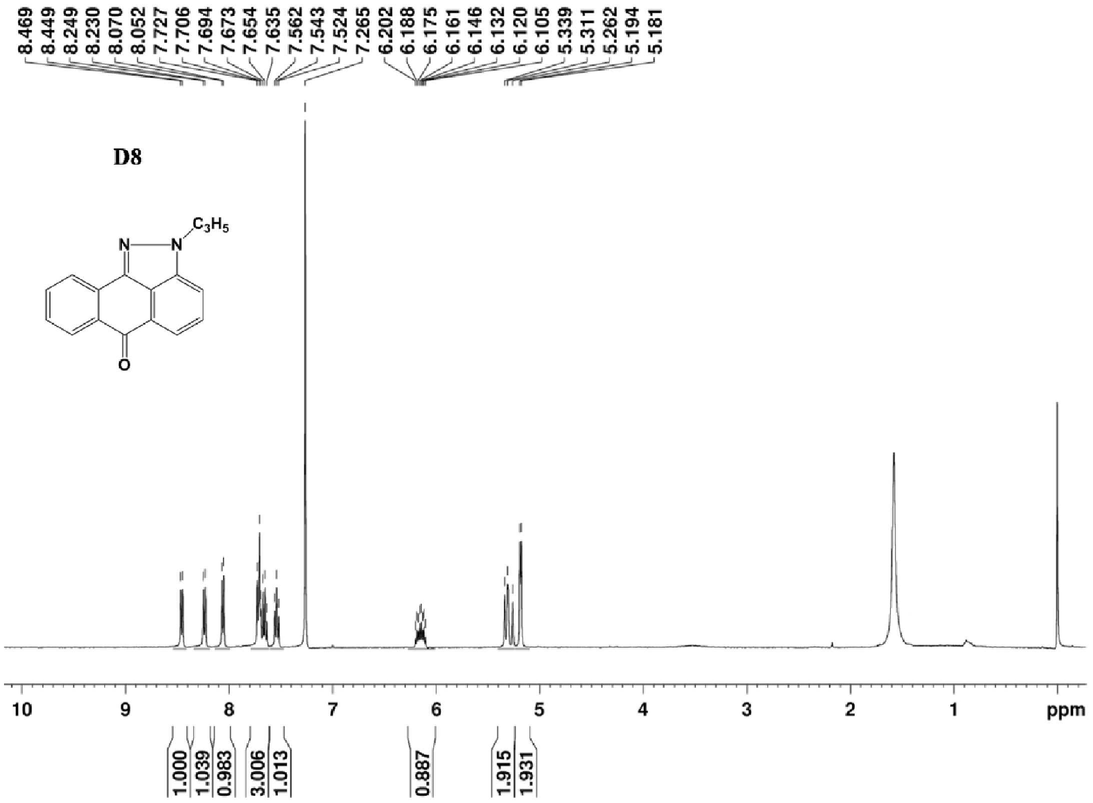

^{1}H NMR spectra of **D8**

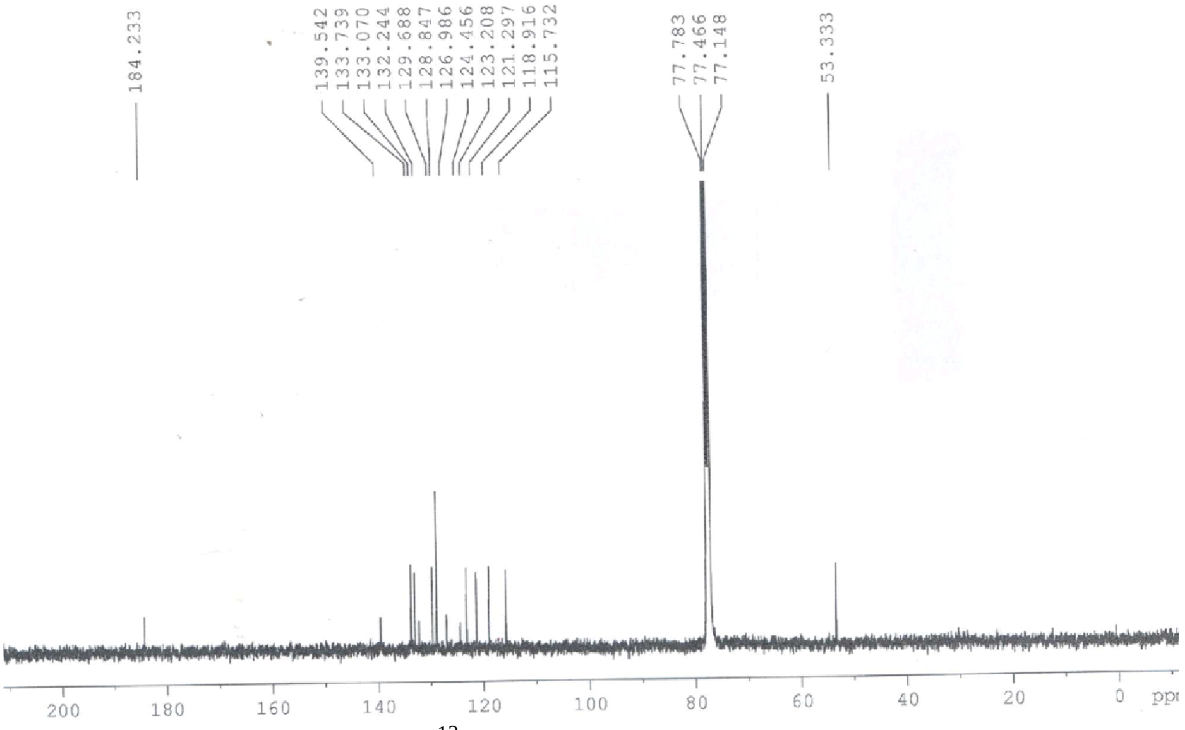

^{13}C NMR spectra of **D8**

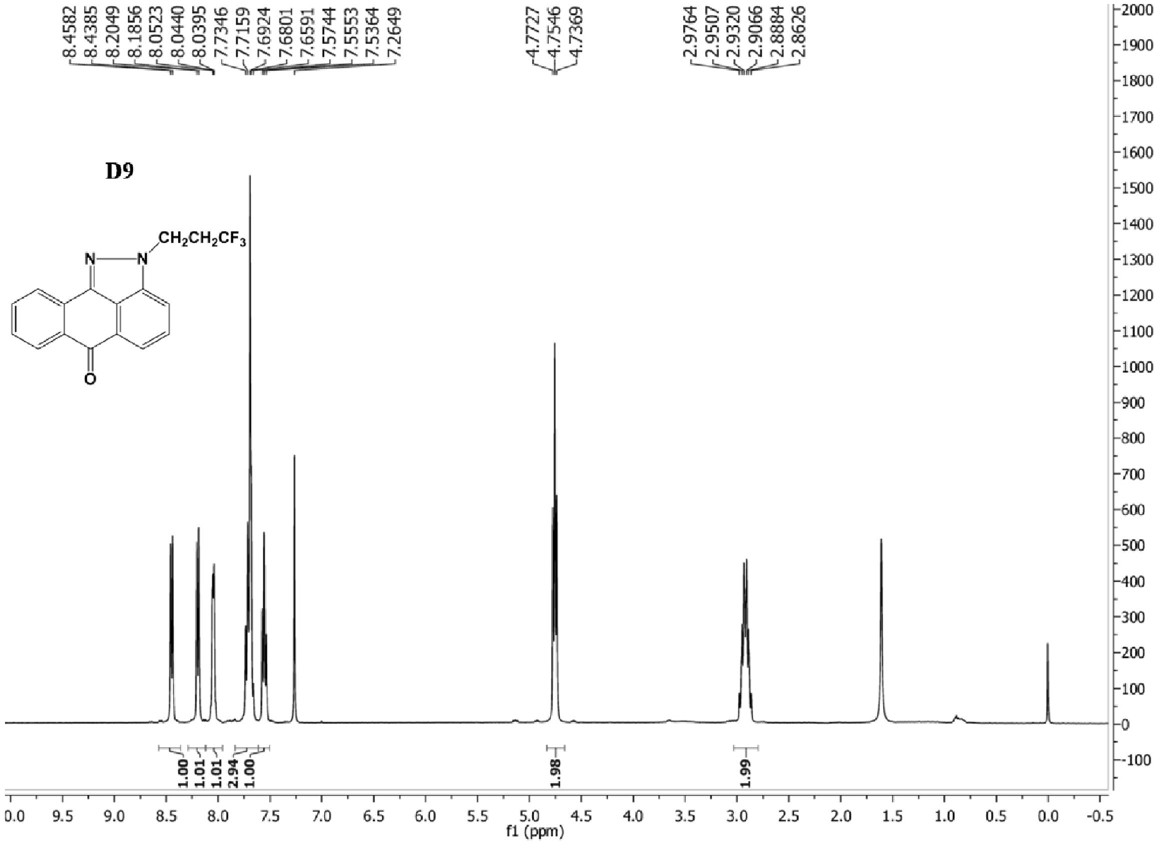

^{1}H NMR spectra of **D9**

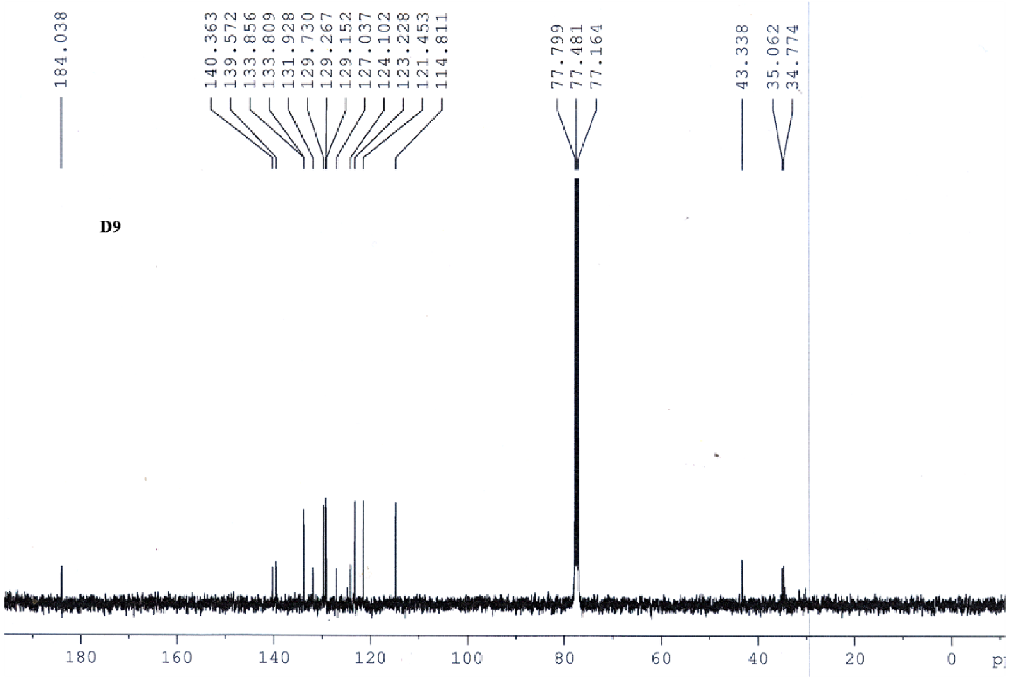

^{13}C NMR spectra of **D9**

HPLC method

Method		
Time (min)	% Methanol	% Acetonitrile
45	30	70

Analytical data for SP60125 and target compounds

Compound	HPLC t_r (min)
SP600125	4.97
SPP1	5.20
SPB1	5.35
D1	4.10
D2	4.20
D3	5.06
D4	6.41
D5	6.63
D6	4.78
D7	5.85
D8	5.52

HPLC Mass Spectra's of anthrapyrazolone and analogues

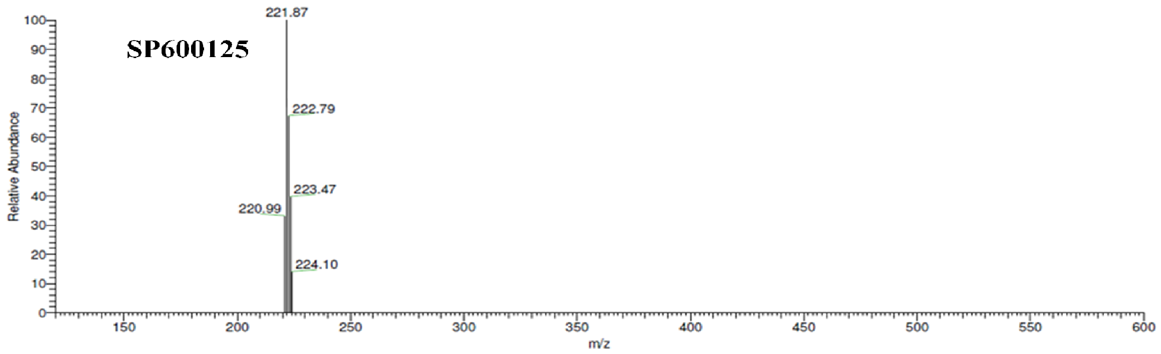

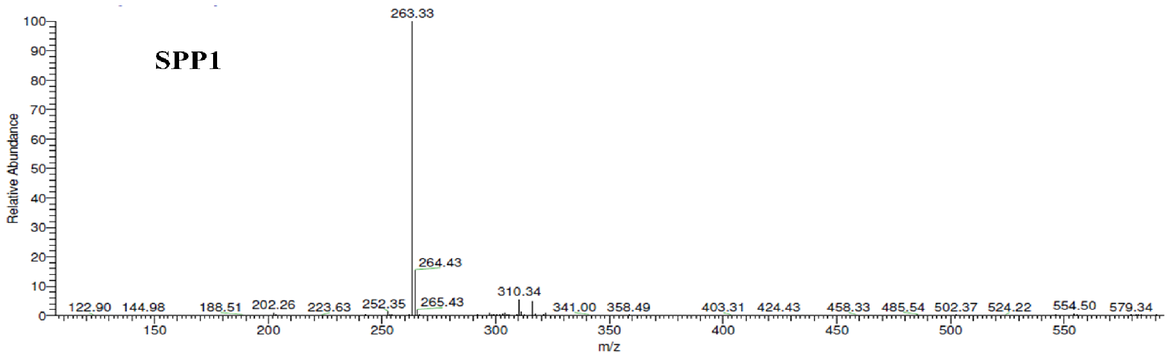

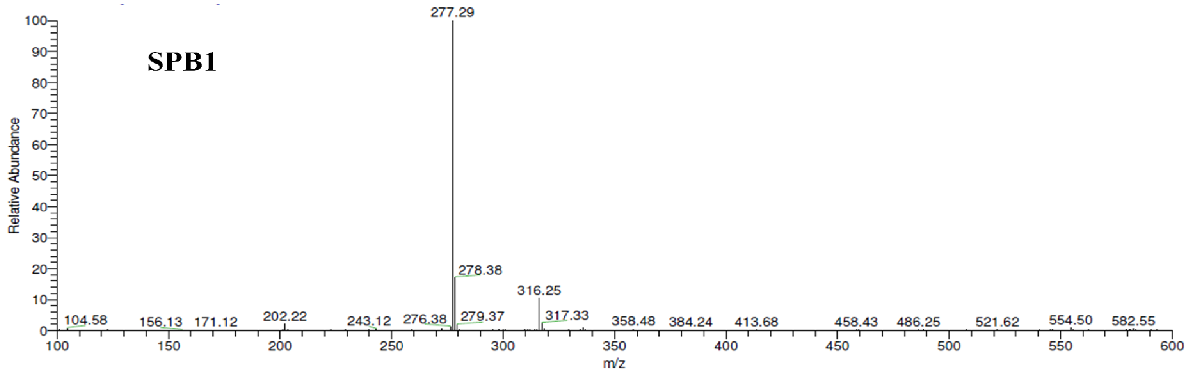
SPB1
Relative Abundance
m/z
277.29
278.38
316.25
104.58
156.13
171.12
202.22
243.12
276.38
279.37
317.33
358.48
384.24
413.68
458.43
486.25
521.62
554.50
582.55

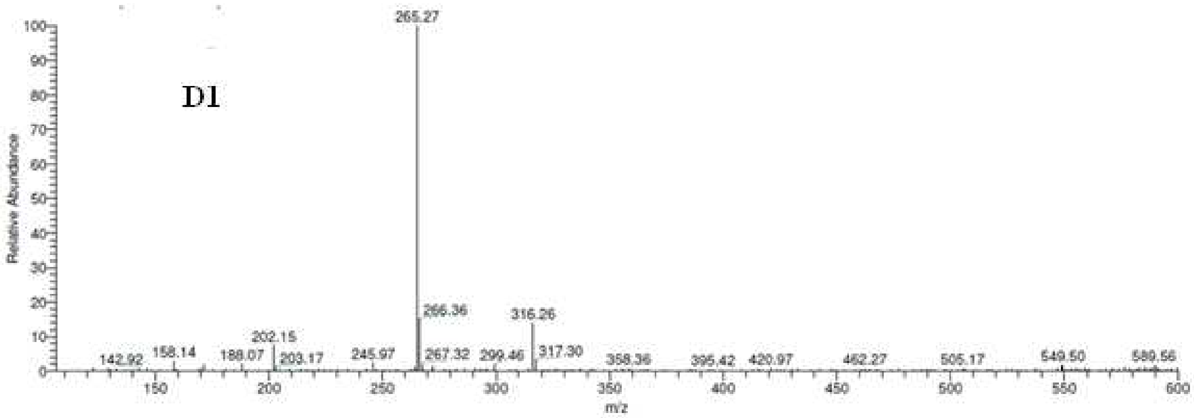
D1
Relative Abundance
m/z
265.27
266.36
316.26
202.15
142.92
158.14
188.07
203.17
245.97
267.32
299.46
317.30
358.36
395.42
420.97
462.27
505.17
549.50
589.56

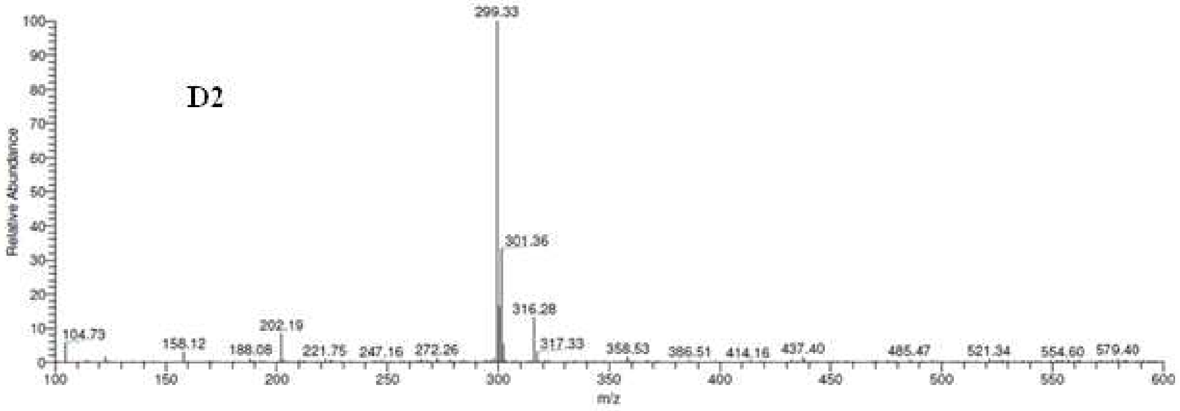
D2
Relative Abundance
m/z
299.33
301.36
316.28
202.19
104.73
158.12
188.08
221.75
247.16
272.26
317.33
358.53
386.51
414.16
437.40
485.47
521.34
554.60
579.40

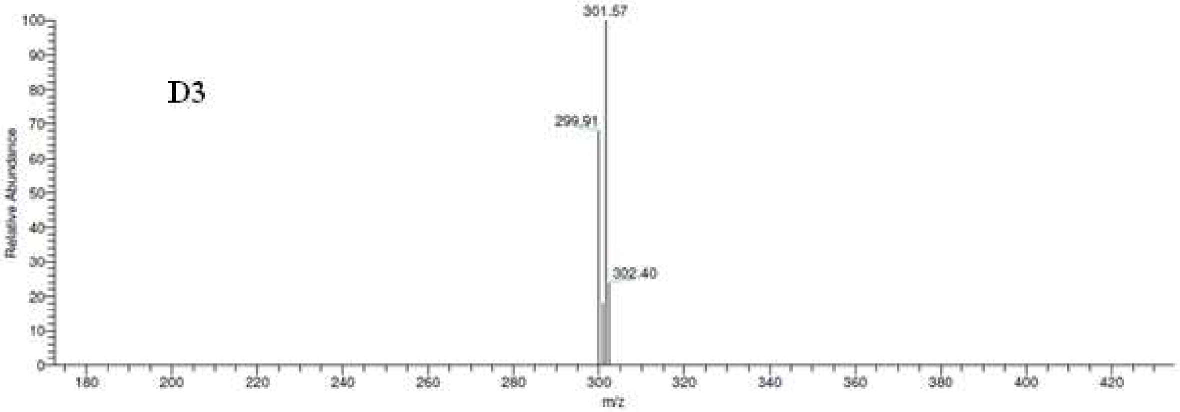
D3
301.57
299.91
302.40
Relative Abundance
m/z

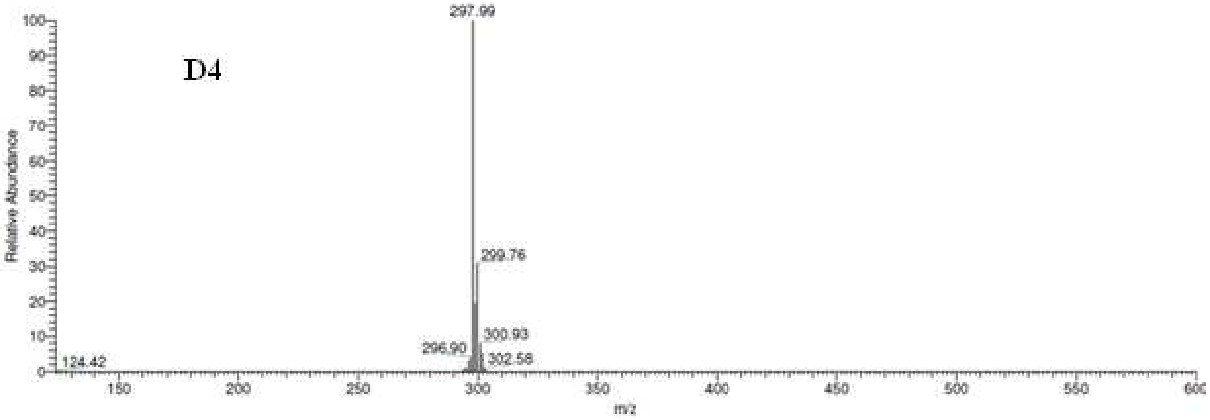
D4
297.99
299.76
296.90
300.93
302.58
124.42
Relative Abundance
m/z

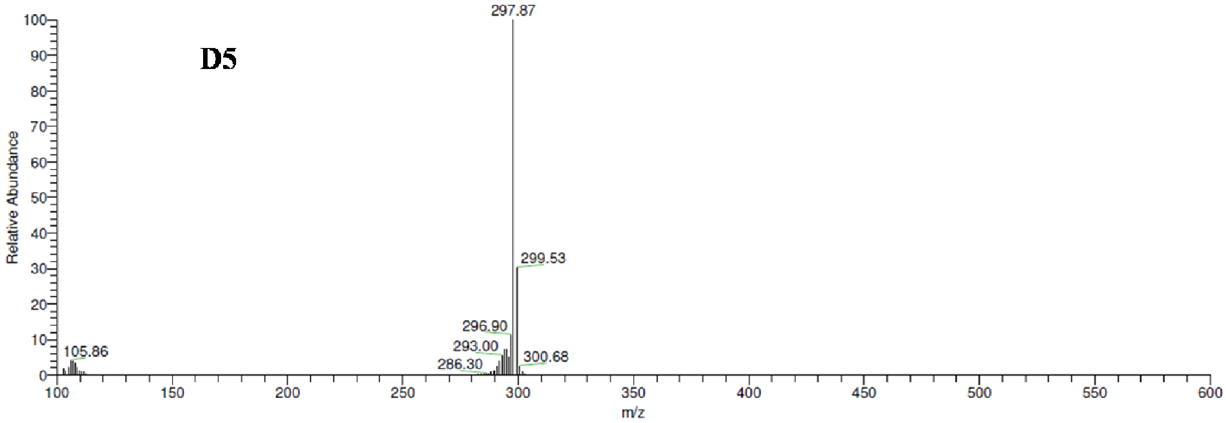
D5
297.87
299.53
296.90
293.00
286.30
300.68
105.86
Relative Abundance
m/z

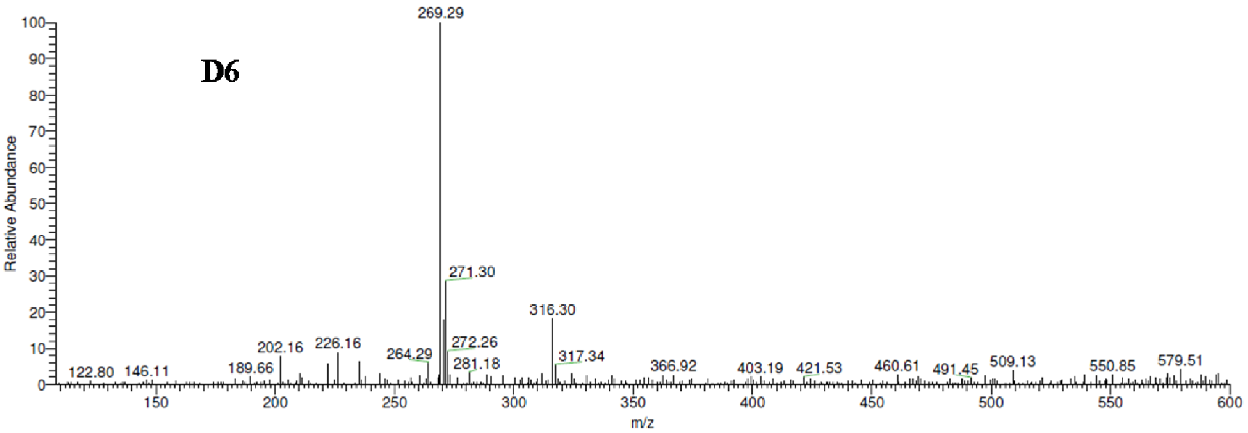
D6
269.29
271.30
316.30
202.16
226.16
264.29
272.26
281.18
317.34
122.80
146.11
189.66
366.92
403.19
421.53
460.61
491.45
509.13
550.85
579.51
Relative Abundance
m/z

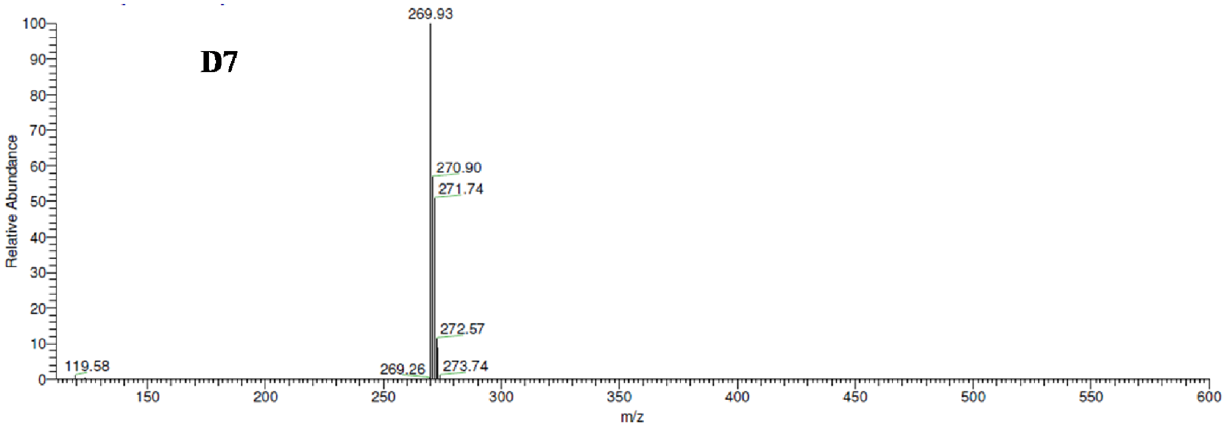
D7
269.93
270.90
271.74
272.57
273.74
269.26
119.58
Relative Abundance
m/z

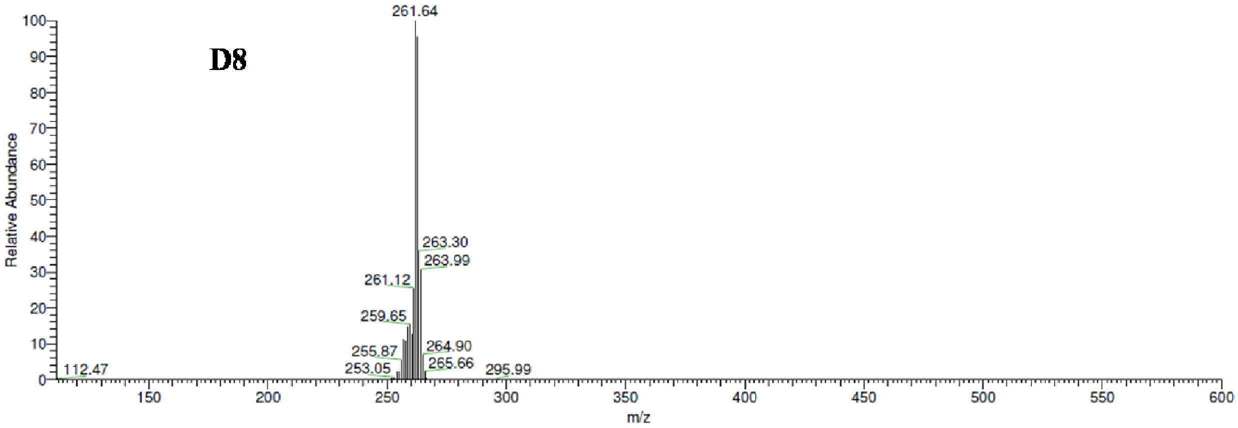
D8
261.64
263.30
263.99
261.12
259.65
255.87
253.05
264.90
265.66
295.99
112.47
Relative Abundance
m/z

References:

(1) Sheldrick, G. M. A short history of SHELX. Acta Crystallogr., Sect. A: *Found. Crystallogr.* **64**, 112-122 (2008).

(2) Farrugia, L. J. WinGX suite for small-molecule single-crystal crystallography. *J. Appl. Crystallogr.* **32**, 837-838 (1999).

(3) Dolomanov, O. V., Blake, A. J., Champness, N. R. & Schröder, M. OLEX: new software for visualization and analysis of extended crystal structures. *J. Appl. Crystallogr.* **36**, 1283-1284 (2003).

(4) Farrugia, L. J. ORTEP-3 for Windows - a version of ORTEP-III with a Graphical User Interface (GUI). *J.Appl. Crystallogr.* **30**, 565 (1997).

(5) Spek, A. Single-crystal structure validation with the program PLATON. *J. Appl. Crystallogr.* **36**, 7-13 (2003).

(6) Macrae, C. F. *et al.* Mercury: visualization and analysis of crystal structures. *J. Appl. Crystallogr.* **39**, 453-457 (2006).

(7) (a) Showalter *et al.* Anthrapyrazole anticancer agents. synthesis and structure-activity relationships against Murine Leukemias *J. Med. Chem.* **30**, 121-131(1987). (b) Bradley, W.& Geddes, K. W. 1 : 9-Pyrazoloanthrone. Part I. Replacement of halogens in derivatives of 1 : 9-pyrazoloanthrone. *J. Chem. Soc.* 1630-1635 (1952). (c) Kim, M. & Wiemer, D. F. EDC-mediated condensations of 1-chloro-5-hydrazino-9,10-anthracenedione, 1-hydrazino-9,10-anthracenedione, and the corresponding anthrapyrazoles. *Tetrahedron Lett.* **45**, 4977-4980 (2004).

(8) Organic Syntheses, Coll. Vol. **6**, p.75 (1988); Vol. **53**, p.13 (1973).

(9) Zhang, R. *et al.* B. B. Design, synthesis and biological evaluation of a novel series of anthrapyrazoles linked with netropsin-like oligopyrrole carboxamides as anticancer agents. *Bioorg. Med. Chem.* **18**, 3974-3984 (2010).

(10) Schuttelkopf, A.W. & Van Aalten, D. M. F. PRODRG: a tool for high-throughput crystallography of protein-ligand complexes. *Acta Crystallogr. D*, **60**, 1355-1363 (2004).

(11) Morris, G. M. *et al.* AutoDock4 and AutoDockTools4: Automated docking with selective receptor flexibility. *J. Computational Chemistry* **30**, 2785-2791 (**2009)**.

(12) Wallace, A. C., Laskowski, R. A. & Thornton, J. M. LIGPLOT: a program to generate schematic diagrams of protein-ligand interactions. *Prot. Eng.* ***8***, 127-134 (**1995)**.

(13) McDonald, I. K. & Thornton, J. M. Satisfying Hydrogen Bonding Potential in Proteins. *J. Mol. Biol.* **238**, 777-793 (1994).
